# Click on Click: Click-Flavone Glycosides Encapsulated in Click-Functionalised Polymersomes for Glioblastoma Therapy

**DOI:** 10.3390/pharmaceutics17060771

**Published:** 2025-06-12

**Authors:** Nuno M. Saraiva, Ana Alves, Ana Isabel Barbosa, Andreia Marinho, Salette Reis, Marta Correia-da-Silva, Paulo C. Costa

**Affiliations:** 1 LQOF—Laboratory of Organic and Pharmaceutical Chemistry, Department of Chemical Sciences, Faculty of Pharmacy, University of Porto, Rua Jorge de Viterbo Ferreira 228, 4050-313 Porto, Portugal; 2 CIIMAR—Interdisciplinary Center of Marine and Environmental Research, University of Porto, Terminal dos Cruzeiros do Porto de Leixões, Avenida General Norton de Matos, S/N, 4450-208 Matosinhos, Portugal; 3 UCIBIO—Applied Molecular Biosciences Unit, MedTech-Laboratory of Pharmaceutical Technology, Faculty of Pharmacy, University of Porto, Rua Jorge de Viterbo Ferreira 228, 4050-313 Porto, Portugal; 4 Associate Laboratory i4HB, Institute for Health and Bioeconomy, Faculty of Pharmacy, University of Porto, Rua Jorge de Viterbo Ferreira 228, 4050-313 Porto, Portugal; 5 LAQV-REQUIMTE—Associated Laboratory for Green Chemistry, Network of Chemistry and Technology, Department of Chemical Sciences, Faculty of Pharmacy, University of Porto, Rua Viterbo Ferreira 228, 4050-313 Porto, Portugal

**Keywords:** click chemistry, glioblastoma, flavones, polymersome, drug delivery

## Abstract

In this study, three new 3,7-dihydroxyflavone (**1**) derivatives with different sugars were designed and synthesised by click chemistry. Click chemistry requires the previously modification of building blocks with azide and alkyne groups and therefore, the 3,7-dihydroxyflavone (**1**) was first converted in 3,7-(prop-2-yn-yloxy)flavone (**2**) and acetobromo-*α*-D-glucose (**3**) was converted into 2,3,4,6-tetra-O-acetyl-*β*-glucopyranosyl azide (**4**). Subsequently, a click reaction was performed via copper-catalysed cycloaddition (CuAAC) between **2** and **4,** as well as between **2** and 2-acetamido-3,4,6-tetra-O-acetyl-2-deoxy-*β*-D-glucopyranosyl (**AG931**) and, **2** and commercial 2-azidoethyl 2,3,4,6-tetra-O-acetyl-*β*-D-glucopyranosyl (**AG358**), resulting in three distinct disubstituted flavone glycosides (**5a**–**5c**). Biological assays performed on L929 fibroblast cell lines and human glioblastoma astrocytoma U-251 cell lines indicated cytocompatibility with fibroblasts and reduced metabolic activity of GBM cells in the presence of compound **5b** and **5c**. To enhance therapeutic effect, improve local drug delivery, and overcome solubility issues of these high molecular weight compounds, the synthesised compounds were encapsulated in polymeric particles (polymersomes, PMs) composed of polylactic acid-polyethylene glycol (PEG-PLA) functionalized, once more by click chemistry, with 0.1 mol% transferrin mimetic (T7—HRPYIAH) peptide. The PMs were prepared by solvent displacement and exhibited stability over 100 days, encapsulation efficiency of 39–93%, and mean size diameters of 120–180 nm. The toxicity assays of the PMs on the U-251 cell line showed a significant decrease in metabolic activity, supporting the potential of this delivery system against GBM. Among the PMs tested, the flavone **5c**-based PM demonstrated the highest efficacy.

## 1. Introduction

Glioblastoma Multiforme (GBM), a grade IV astrocytoma commonly known as Glioblastoma, is the most aggressive, invasive type and accounts for almost 80% of all Central Nervous System tumours [1,2,3,4,5,6]. GBM is considered a rare disease, and the current treatment includes surgery, radiotherapy, and/or chemotherapy [1,7]. GBM presents a significant intratumorally heterogeneity, high mitotic activity, tissue necrosis, vascular proliferation, resistance to apoptosis, and penetration into adjuvant cells [8]. This rapid growth in deep brain areas complicates local accessibility and the easiness of diffusion makes the tumour resection after surgery almost impossible [9,10]. Therefore, this cancer is associated with a poor prognosis: The median survival rate is 12 months [4,5,11,12] as patients invariably relapse [13]. After five years of post-diagnosis, the survival rate is less than 5% [14]. The worldwide incidence is less than 0.01% and is 1.6 times greater in men than women. As for the current therapeutic agents, temozolomide is the first-line chemotherapeutical drug in the use of GBM treatment [15,16,17,18,19].

The biological challenges of therapy are significant. Firstly, the ineffectiveness of antiproliferative therapies can be partly attributed to the discovery of tumour heterogeneity. This dynamic heterogeneity enables resistance to combination oncology, with therapy-resistant subclones forming as the tumour grows [20,21]. Secondly, treatment resistance and tumour recurrence in GBM may be linked to its microenvironment [22,23,24,25,26,27]. Finally, the blood–brain barrier (BBB) consists of a layer of non-fenestrated capillary endothelial cells interconnected through a network of intercellular junctions [28]. In both primary and metastatic brain tumours, the integrity of the BBB is compromised, leading to the formation of the brain–tumour barrier (BTB). A leaky BBB/BTB implies that the membrane no longer restricts drug delivery, affecting the effectiveness of treatments [29]. Emerging evidence [30,31,32,33,34] suggests that the heterogeneous breakdown of the BBB makes it challenging to achieve uniform drug concentrations inside the tumour.

Flavonoids are a large and wide family of polyphenolic metabolites derived from plants [35,36,37,38]. Flavonoids have a unique structure: a three-ring base consisting of two benzene rings linked either between a pyran heterocyclic ring or a three-carbon liker forming a fifteen-carbon skeleton (C6-C3-C6 system). Scientists have found over five thousand flavonoids from a plethora of plants [35,36,37,39]. There is evidence that many natural flavonoids possessed anticancer activity. For example, isoflavone Genistein promotes breast cancer cell arrest and induces Reactive Oxygen Species dependent apoptosis [40]. Daidzein induces apoptosis in breast cancer cells [41]. Flavanone Hesperetin triggers apoptosis in bladder carcinoma [42], oesophageal cancer [43], hepatocellular carcinoma [44], and human breast carcinoma [45]. Flavonoids such as Quercetin, Myricetin, Genistein, Delphinidin, Luteolin, Apigenin, Baicalein, Galangin, or even Diosmetin can decrease the level of proliferation and/or invasion of GBM cells [46]. Synthetic flavonoids [35,36,47] have also been reported for their antitumoral activity. From the several synthetic flavonoids reported with antitumor activity, two acetic acid flavonoids have been reported to have activity against advanced non-small cell lung cancer [48].

Several bioactive synthetic flavonoids were also reported by our group [49,50,51,52], and among them, an acetoglycosyl flavonoid was shown to have an anti-proliferative effect in human cancer cell lines, namely GBM [53].

In this study, we designed three new di-acetoglycosyl triazole-linked flavones. We describe their synthesis, structure, and anti-proliferative properties for the first time. By adding a triazole ring between the flavone and acetylated glycosides, we improve their metabolic stability compared to the more common *O*-glycosidic bonds [54]. To overcome their limited solubility, we encapsulated these compounds in polymersomes (PMs), using transferrin-triazole-linked PEG-PLA copolymers [55], while enhancing their potential for targeting delivery. PMs are spherical and hollow nanosystems composed of amphiphilic copolymers that can encapsulate hydrophilic and hydrophobic drugs, individually or at the same time [56]. These drug delivery systems (DDS) have more content retention [57], have superior functionalisation choices [58], higher bioavailability and biodegradability, and have the possibility of cargo release induced by stimuli, compared to others [51,54,58].

## 2. Results and Discussion

### 2.1. Synthesis and Structure Elucidation

Three new di-glycosyl flavones bearing a 1,2,3-triazole moiety and different sugar moieties were synthesised in this study.

There has been a great interest in the synthesis of 1,2,3-triazole functional groups since these moieties are more than just linkers. They have some favourable physicochemical properties and have shown importance to biological activity [59,60]. This strategy generates many biologically potential compounds [61,62,63,64] since hundreds of synthetic 1,2,3-triazole-linked flavonoid analogues have been reported to have antitumor activity [52]. For the chemical synthesis of these triazole-linked flavones, the 2022 Nobel prize “click” chemistry was chosen. Sharpless and co-workers [65] enormously impacted chemistry philosophy by discovering “click” reactions. Click chemistry is a class of nearly perfect chemical reactions that are effective in terms of atom economy, stereo-specificity, wide scope, and almost all properties that today are called the green chemistry principles. The reaction is enormously selective since it only occurs when azide and alkyl groups are present. The copper-catalysed azide–alkyne cycloaddition (CuAAC) is a variant of the classical thermal Huisgen 1,3-dipolar cycloaddition and was described by Sharpless as the ‘cream of the crop’ of click chemistry [66,67]. By using copper (CuSO_4_) and sodium ascorbate, the energy necessary for the activation barrier is decreased significantly, making the reaction possible to proceed at room temperature and in aqueous or organic solvents, leading to a 1,4-disubstituted triazole [68].

**Scheme 1:**
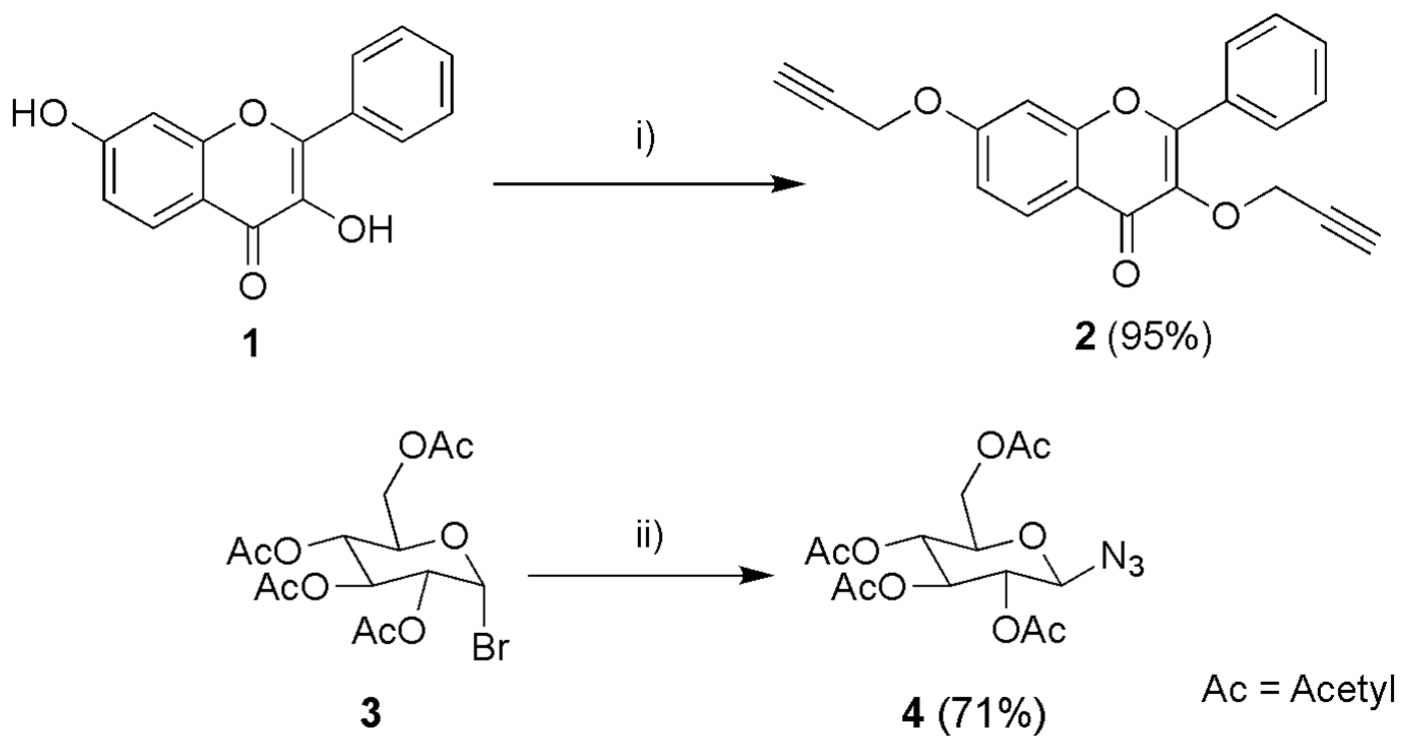
Synthesis of 3,7-(prop-2-yn-yloxy) flavone (**2**) and 2,3,4,6 tetra-O-Acetyl-*β*-D-glucopyranosyl azide (**4**). (i) Propargyl bromide, Tetrabutylammonium hydrogen sulphate (TBAHS), Cs_2_CO_3_, acetone, 60 °C, 8 days; (ii) NaN_3_, acetone:water, room temperature, 4 h.

To achieve these flavones, the flavone **2** and the glycoside **4** were firstly synthesised (Scheme 1). The 3,7-dihydroxyflavone (**1**) propargylation was carried out in basic conditions, with acetone as solvent due to its polar aprotic characteristics. The usage of TBAHS was to help solubilise the negative charges of the phenoxide ion. This reaction proceeded as in previous work from our group; it was inspired by a propargylation reaction on polyphenols [69] and provided a yield of 95% of compound **2**.

A simple and effective glycosyl-azide synthesis was carried out by a reaction of glycosyl-halide with sodium azide in aqueous acetone as solvent. It has been shown that the combination of water-acetone as solvent is more efficient than dry DMF, caused by poor solubility of NaN_3_ [70]. Dissolving NaN_3_ in water increases the concentration of the azide ions and thus the reaction rate. After the acetone evaporated, the pure solid precipitated from the water. The reaction provided a yield of 71%.

**Scheme 2:**
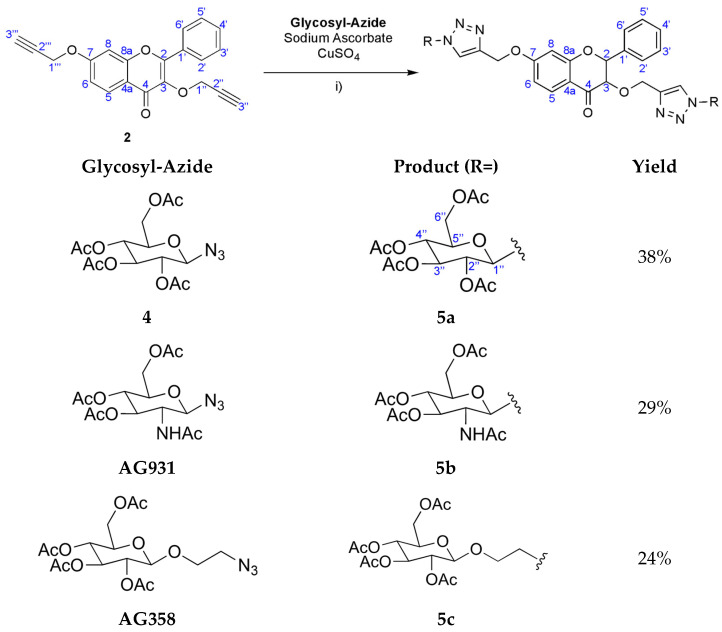
Synthesis of flavonoids **5a**–**5c**. (i) **5a**: **4**, THF: water, room temperature, 24 h; **5b**: **AG931**, THF: water, room temperature, 2 h; **5c**: **AG358**, THF: water, room temperature, 2 h. Ac = Acetyl (COCH_3_).

Compounds **5a**–**c** were synthesised by CuAAC. The reactions involved the previously formed flavone (**2**) together with three similar glycosyl-azide substrates: 2,3,4-tetra-O-acetyl-*β*-glucopyranosyl azide (**4**), commercial 2-acetamido-3,4,6-tetra-O-acetyl-2-deoxy-*β*-D-glucopyranosyl (**AG931**), and commercial 2-azidoethyl 2,3,4,6-tetra-O-acetyl-*β*-D-glucopyranosyl (**AG358**). These reactions were performed in the presence of copper sulphate (CuSO_4_) and sodium ascorbate in a non-homogeneous mixture of water and THF (1:4) at room temperature (Scheme 2).

After the THF evaporated, an impure white solid precipitated in the remaining water in the case of **5a** and **5b** syntheses. Therefore, by using chromatographic preparative TLC plates (5:5 CHCl_3_—Acetone), **5a** was obtained in 35% yield and **5b** in 29% yield. In the case of **5c** synthesis, no solid was formed from water. This water solution was then extracted with AcOEt and the organic phase purified by crystallisation from MeOH/CH_2_Cl_2_, where **5c** was obtained in 24% yield.

However, it was not the first time that chromatographic procedures together with the low yield obtained and the presence of by-products were described when using click chemistry [52,65,71].

For the flavonoid building block (**2**), the alkyne group could be easily identified by a strong band of the triple CC bond at 2107 cm^−1^, and another band at 3274 cm^−1^ from the alkyne group (≡C-H). By ^1^H NMR spectrometry, the two proton signals from the two alkynes functional groups (≡C-**H**) could be observed (*δ*H-3‴ 3.68 s, *δ*H-3″ 3.47 s). Similarly, by ^13^C NMR spectrometry, the four carbon signals from the two alkenes (**C**≡**C**) were also observed (*δ*C-3‴ 79.3, *δ*C-3″ 79.1, *δ*C-2″ 78.7, *δ*C-2‴ 78.3). For the glycosyl-azide building block (**4**), the presence of the azide could be observed by the characteristic strong band at 2119 cm^−1^.

**Figure 1:**
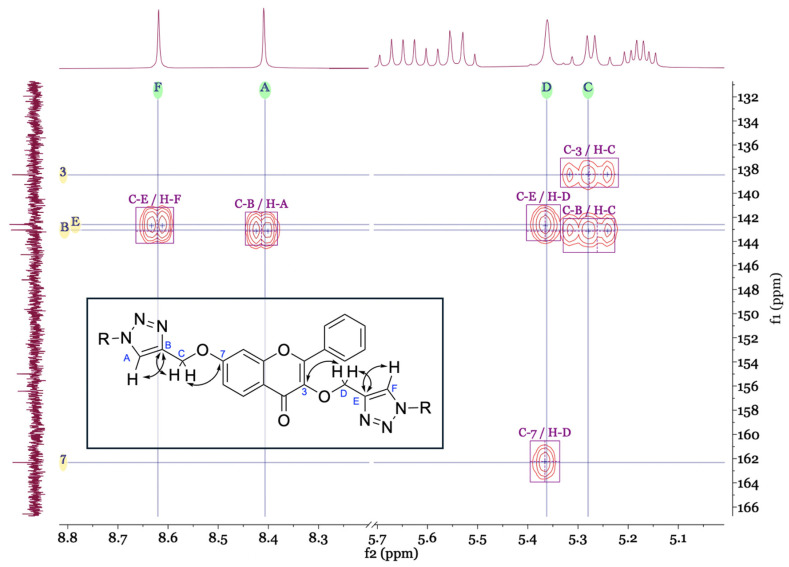
Main connectivities found in HMBC for the identification of the triazole ring positions.

The NMR spectra (see Supplementary Material) of the newly synthesised compounds **5a**–**5c** showed characteristic signals for the flavone scaffold and the glycosyl moieties. Additionally, signals of the triazole proton (*δ*H 8.62–7.88, s) and carbons (*δ*C 143.1–141.6 and 125.2–123.8) were observed by NMR. The placement of the triazole ring in these compounds was confirmed by the heteronuclear multiple bond correlation (HMBC) spectra. Special nomenclature for this correlation attributions’ were used. These spectra showed a correlation between the proton signals A (-C=C*H*-triazole) and C (-O-C*H*_2_-triazole) with carbon B signal (-*C*=CH-triazole), and carbon B with the C-7 signal. The same was observed for the other triazole ring with two bridges of correlations between the proton signals D and F with carbon E signal, and proton D signal with C-3 signal (Figure 1). These correlations were observed for the three di-glycosyl flavones.

The molecular formula of **5a**–**5c** was also confirmed by HRMS as C_49_H_52_N_6_O_22_ (1099.288), C_49_H_54_N_8_O_20_ (1097.319) and, C_53_H_60_N_6_O_24_ (1187.338), respectively.

Comparing the IR spectrum of **5a** with **2**, the spectrum of **5a** has a second strong band due to carbonyl groups of the acetyl groups at 1751 cm^−1^ and other at 1227 cm^−1^ typical of C-O of the acetyl groups. Also, it is visible that the characteristic strong band of the triple CC bond (at 2107 cm^−1^) disappears, underscoring the reaction success. This is also visible in the IR spectrum of **5b** and **5c**: here, we see the new acetyl C=O bands at 1747 cm^−1^ and 1750 cm^−1^, respectively. For the acetamido **5b** compound, a strong band at 1621 cm^−1^ was observed, characteristic of the amide C=O stretch.

### 2.2. Cytotoxicity of Free Compounds

Acetylated flavonoids **5a**–**5c** were evaluated for their in vitro growth inhibitory by the (3-(4,5-dimethylthiazol-2-yl)-2,5-diphenyltetrazolium bromide) MTT assay. MTT assay measures cellular metabolic activity as an indicator of cell viability and proliferation. This colorimetric assay is based on the chemical reduction in the yellow tetrazolium salt (MTT) to purple formazan crystals by metabolically active cells.

**Figure 2:**
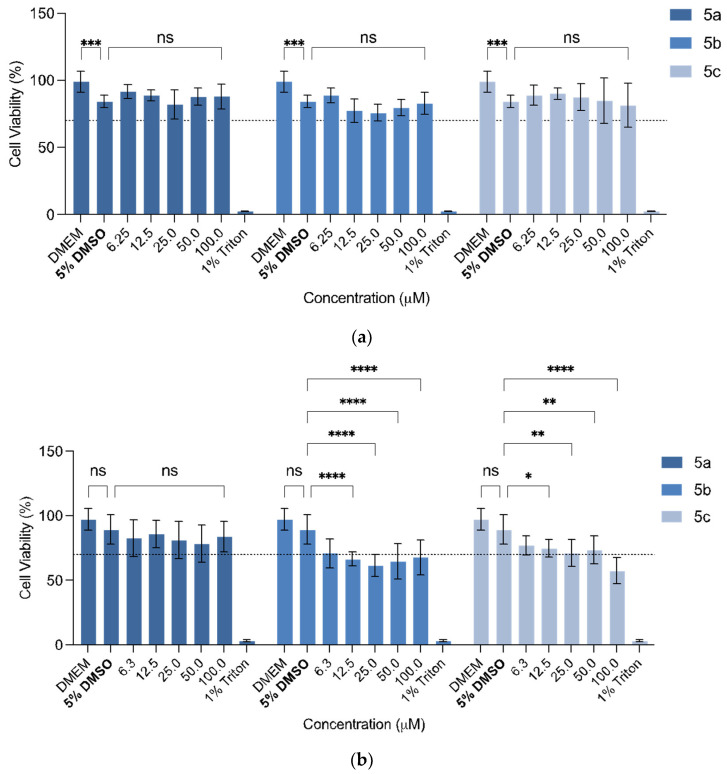
Cell viability (%) effect on (**a**) the fibroblast L929 cell line and (**b**) the human glioblastoma astrocytoma U-251 cell line of free synthesised compounds. Each value represents a total of *n* = 3 replicates of *n* = 3 independent assays (a total of 9 replicates). DMEM was used as a positive control and 1% Triton X-100 as a negative control. * (One asterisk): *p*-value <0.05; ** (Two asterisks): *p*-value < 0.01; *** (Three asterisks): *p*-value < 0.001; **** (Four asterisks): *p*-value < 0.0001.

The cytotoxicity of compounds on the fibroblast L929 cell line (Figure 2a) was first evaluated according to the procedure described by the International Organization for Standardization (ISO 10993-5:2009) [72].

The presence of 5% DMSO had significant effect on cell viability (CV) of the L929 cell line (*p* = 0.0003, Figure 2). Considering the cytotoxicity of the compounds in this cell line, some significant changes in the CV percentages were observed; however, none of them decreased the CV below 70% (which is the safety threshold, according to the ISO 10993-5:2009) (Figure 2a). None of the compounds showed significant viability changes, proving that their use is safe up to 100 µM (**5a**: *p* > 0.9999; **5b**: *p* = 0.4695; **5c**: *p* = 0.2876).

**Figure 3:**
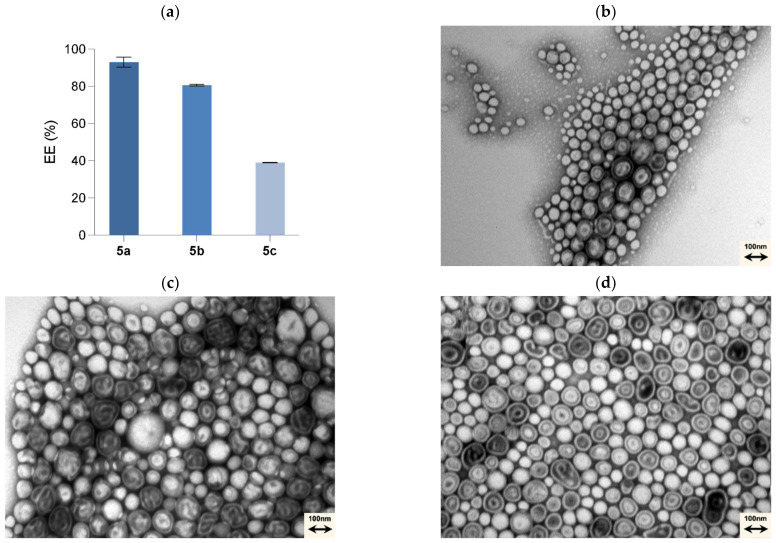

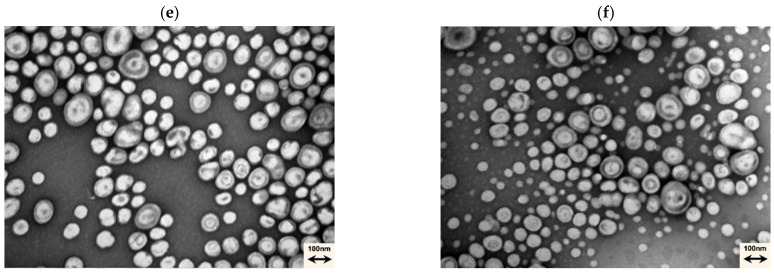
Entrapment efficiency (EE%) of the compounds synthesised (**a**) and TEM images of the formulations 0 mol% **T7-PEGPLA** (control) (**b**), 0.1 mol% **T7-PEGPLA** (control) (**c**), 0.1 mol% **T7-PEGPLA-5a** (**d**), 0.1 mol% **T7-PEGPLA-5b** (**e**), and 0.1 mol% **T7-PEGPLA-5c** (**f**).

The same compounds were also tested, with a similar procedure, against the human glioblastoma astrocytoma U-251 cell line (Figure 3b). The presence of 5% DMSO did not affect the CV of the U-251 cell line. An anticipated outcome of these studies would be the decrease in the CV vs. concentration in this cell line in contrast to the first cell line tested. Compound **5b**, with the acetylated *N*-acetyl glucosamide, significantly decreased the CV across all concentrations (*p* = 0.0018, for 6.3 µM). However, the CV did not decrease below 50%, even at the highest concentration tested (100 µM). Regarding compound **5a**, there was no significant differences across all concentrations, and compound **5c**, the only statistically significant difference against this cell line, was at >12.5 µM (*p* = 0.0248). Here, once more, the CV did not decrease below 50%.

### 2.3. Formation and Characterisation of Loaded and T7-Functionalised PMs

Transferrin is a plasma protein that transports iron and binds to receptors overexpressed on the BBB due to the high iron demand for intermediary metabolism [73]. These receptors are also overexpressed in several tumour cells [74]. To access this functionality, T7 (HAIYPRH) peptide, a targeting ligand that can specially bind to transferrin receptors was employed to be part of the PMs delivery system. This peptide is a combination of seven amino acids (His-Ala-Ile-Tyr-Pro-Arg-His) and was commercially functionalised to have an alkyl end-group. T7-functionalised PEG_20_PLA_112_ (**T7-PEGPLA**) polymer was prepared as reported in Ref. [55].

The same solvent displacement method was employed in the assembly of these functionalised copolymers. **T7-PEGPLA** and **PEGPLA** were mixed with a molar ratio of 0,1%. The mean diameter and the PDI of the five formulations of **T7-PEGPLA**, mixed with **PEGPLA** and loaded with **5a**–**5c**, were evaluated by DLS (Table 1). The zeta potential (ζ) was also measured.

In a statistical analysis of the mean diameter, the empty PMs’ formulations (0 mol% and 0.1 mol%) did not show any statistical difference (*p* = 0.9993), proving that the presence of the **T7-PEGPLA** does not alter the morphology of the particle.

Regarding the loaded PMs, the biggest increase in size was with the 0.1 mol% **T7-PEGPLA**-**5a** formulation, from a mean of 122.7 to 184.4 nm (*p* < 0.0001). The 0.1 mol% **T7-PEGPLA**-**5b** formulation had a mean of 161.6 nm (*p* = 0.0010), whereas the 0.1 mol% **T7-PEGPLA**-**5c** formulation had a mean of 153.2 nm (*p* = 0.0030). These differences in size can be correlated to the entrapment efficiency (Figure 3a) of each formulation, given that the empty formulations (0 mol% and 0.1 mol%) show similar mean diameters and PDIs. Compound **5a** had the biggest entrapment efficacy (93 ± 2.73%), followed by **5b** (80 ± 0.49%), and finally **5c** (39 ± 0.12%). Higher EE% seems to increase more the mean diameter than a lower EE% formulation.

Also, by TEM analysis (Figure 3b–f), it is possible to visually affirm that no morphological change occurs.

**Figure 4:**
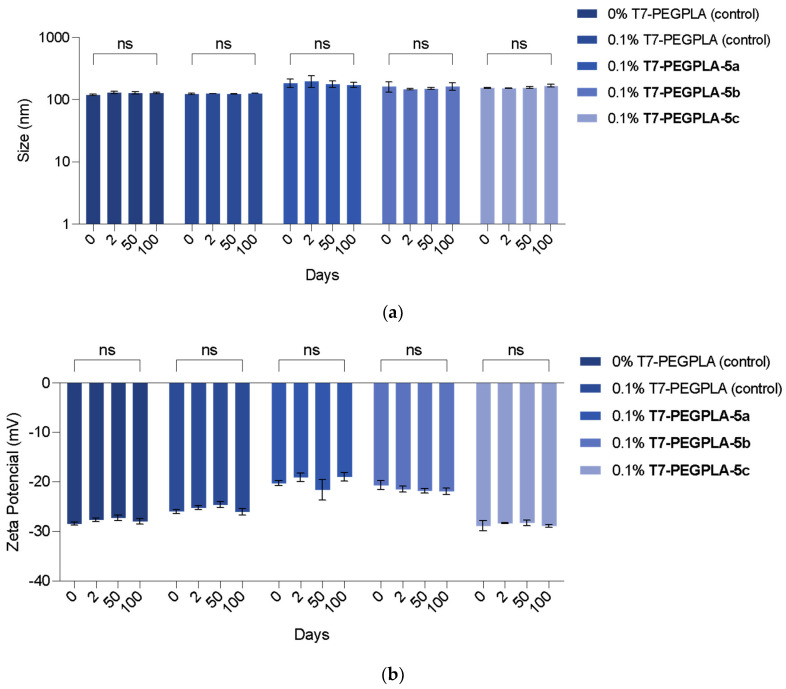
Effective mean diameter (**a**) and zeta potential (**b**) of functionalised PMs formulations stored at 4 °C for 100 consecutive days (each value represents a total of *n* = 3 replicates). The Size (nm) *y*-axis is presented in a logarithmical (log_10_) scale.

The functionalised PMs were stored over time at 4 °C, their stability was determined by DLS and Zeta potential, and showed no significant changes for 100 days, indicating the strong stability of the prepared diblock copolymers’ PMs and the stability of the system with the entrapped compounds (Figure 4).

### 2.4. Anti-Glioblastoma Activity of Compounds Entrapped in Functionalised PMs

**Figure 5:**
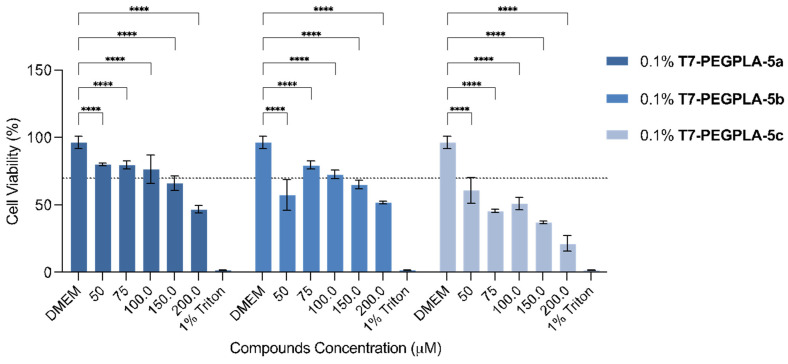
Cell viability (%) effect on the human glioblastoma astrocytoma U-251 cell line of loaded PMs with synthesised flavones. Each value represents a total of *n* = 3 replicates of *n* = 3 independent assays (a total of 9 replicates). DMEM was used as the positive control and 1% Triton X-100 as a negative control. **** (Four asterisks): *p*-value < 0.0001.

The three formulations containing the entrapped flavonoids were evaluated for their in vitro growth inhibitory, also by the MTT assay (Figure 5). The usage of 5% DMSO was no longer needed given that the compounds could be administered in an aqueous media with the help of the PMs’ system. The assay was carried out taking considering the compounds’ concentration inside the PMs, given that the intrinsic toxicity of this PMs was already proven to not be significant [55]. There was a visible effect of the exposure of the U-251 cell line to the prepared PMs. The PMs’ system with compounds **5a** and **5b** only decreased the cell viability of the U-251 cell line below 50% at 200 µM, resulting in these systems only exhibiting significant effects at higher concentrations. For the PMs with compound **5c**, with the ethyl linker, almost all concentrations tested decreased the cell viability below the 50%, with the highest concentration resulting in only 21% of GBM cell line viability. Compared with the free compounds (Figure 2b), it is possible to see that, in the same dose, this compound can achieve a higher effect on the cell viability. Therefore, this compound, by having a substitution of a 2-azidoethyl in the anomeric carbon, proved to be the most promising against the U-251 cell line of the three analogues.

By entrapping the flavonoids into PMs, we were able to apply higher dosages to the cells and achieve more effects compared to the free compounds, highlighting the higher bioavailability that these systems provide for the entrapped compounds.

Many published studies already involve the encapsulation of drugs into PMs. Some of the examples of these drugs already encapsulated are the antitumoral Doxorubicin [75,76,77,78,79,80,81,82,83,84,85,86,87,88,89] and Paclitaxel [84,90,91,92], and others, such as Azobenzene [93], Hypericin [93], Avidin [94], Rhodamine B [82,95,96], Calcein [97], and Insulin [98,99].

However, none of them are in clinical trials or in therapeutics [100,101,102].

## 3. Materials and Methods

### 3.1. General Information

All solvents used were from Sigma-Aldrich Co. (St. Louis, MO, USA) and Thermo Fisher Scientific (Waltham, MA, USA). Sodium Azide (NaN_3_, S2002 and 71290), (+)-sodium-L-ascorbate (A7631), 3,7-dihydroxyflavone (**1**, 419826), propargyl alcohol (P50803), sodium cyanoborohydride (NaBH_3_CN, 156159), glucose (101175P), glucosamine (G4875), and silver carbonate (AgCO_3_, 179647) were acquired from Sigma-Aldrich Co. Acetobromo-*ɑ*-D-glucose (**3**, L04151.18), tetrabutylammonium hydroxide (TBAHS, AC168380250), caesium carbonate (Cs_2_CO_3_, 12887), and propargyl bromide (AC131482500) were acquired from Alfa Aesar (Thermo Fisher Scientific). 2-acetamido-3,4,6-tetra-O-acetyl-2-deoxy-*β*-D-glucopyranosyl azide (AG931) and 2-azidoethyl 2-acetamido-3,4,6-tri-O-acetyl-2-deoxy-*β*-D-glucopyranosyl (AG904) were acquired from Synthose Inc. (Concord, ON, Canada). Boron trifluoride ethyl etherate (BF_3_.OEt_2_, B0527) were acquired from Tokyo Chemical Industry Co., Ltd. (TCI, Tokyo, Japan). Copper (II) sulphate (CuSO_4_, CL00.1127.1000) was acquired from Chem-Lab NV (Zedelgem, Belgium).

The reactions were monitored by Thin Layer Chromatography (TLC) aluminium sheets, silica gel 60 with a fluorescent indicator (ALUGRAM^®^ Xtra SIL G UV254) from Macherey–Nagel GmbH & Co. KG. (Düren, Germany). The purifications by preparative TLC were performed with 20 × 20 cm glass plates covered with silica gel G/UV254 with a fluorescent indicator (816320.5) from Macherey–Nagel GmbH & Co. KG. High-performance liquid chromatography (HPLC) was performed on a Dionex Ultimate 3000 (Thermo Scientific, Darmstadt, Germany), with AcclaimTM 120 C18 (100 × 4.6 mm) column with a particle size of 5 μm, from Thermo Fisher Scientific (Bremen, Germany), detector 220 nm, 265 nm, and 330 nm, and 1 mL/min isocratic flow. IR spectra were obtained in KBr microplates in a Fourier transform infrared spectroscopy (FTIR) spectrometer Nicolet iS10 from Thermo Scientific with Smart OMNITransmisson accessory (Software OMNIC 8.3) (cm^−1^). The ^1^H and ^13^C-NMR spectra were obtained at the CEMUP (Centro de Materiais da Universidade do Porto), Porto, Portugal, on a Bruker Ascend III 400 instrument (^1^H: 400.14 MHz; ^13^C: 100.63 MHz). The ^13^C-NMR assignments were made by bidimensional HSQC and HMBC experiments (long-range C, H coupling constants were optimised to 7 and 1 Hz). The chemical shifts are expressed in ppm values relative to tetramethylsilane (TMS) as an internal reference, and coupling constants are reported in hertz (Hz). The assignment’s abbreviations are as follows: doublet (d), double doublet (dd), double double doublet (ddd), double double double doublet (dddd), double quartet (dq), double triplet (dt), multiplet (m), singlet (s), triplet (t), and triple doublet (td). HRMS spectra were obtained at the CEMUP (Centro de Materiais da Universidade do Porto), Porto, Portugal, on the LTQ Orbitrap XL hybrid mass spectrometer (Thermo Fischer Scientific, Bremen, Germany).

Cell reagents were purchased from Gibco (Invitrogen Corporation, Life Technologies, Cramlington, UK). Cell reagents (Dulbecco’s Modified Eagle’s Medium (DMEM), Fetal Bovine Serum (FBS), Penicillin–Streptomycin (Pen Strep), and Trypsin EDTA) were provided by VWR (Leuven, Belgium), Biowest (Nuaillé, France) and Gibco (Life Technologies, S.A. (Madrid, Spain)). The 929 Fibroblast cell line was obtained from the ‘European Collection of Authenticated Cell Cultures’ (ECACC, UK), and the U-251 Glioblastoma Astrocytoma cell line was kindly donated by Dr Salette Reis. In figures, different asterisks (*) in the same sample represent significant differences: *p*-value > 0.05; * (One asterisk): *p*-value < 0.05; ** (Two asterisks): *p*-value < 0.01; *** (Three asterisks): *p*-value < 0.001; **** (Four asterisks): *p*-value < 0.0001. All the statistical analyses were performed with GraphPad Prism 10 for macOS (Version 10.1.1, 21 November 2023).

### 3.2. Chemistry

#### 3.2.1. Synthesis of 3,7-(Prop-2-yn-yloxy)flavone (**2**)

3,7-Dihydroxyflavone (**1**, 2.00 g, 7.87 mmol), TBAHS (26.71 g, 78.67 mmol, 5 equiv/OH), and Cs_2_CO_3_ (38.45 g, 118.00 mmol, 7.5 equiv/OH) were added in acetone (250 mL). The mixture was refluxed for 1 h at 60 °C until maximum solubilisation. A propargyl bromide solution (80% wt.% in toluene, 21.35 mL, 196.66 mmol, 12.5 equiv/OH) was added and left stirring for eight days until the starting material **1** had been consumed. The product showed a purple fluorescence (normal-phase analytical TLC, 95:5 CHCl_3_—MeOH, *R_f_* = 0.80). The mixture was filtered and washed with acetone, providing a brown filtrate that, after being evaporated under reduced pressure, provided a residue oil that was purified by crystallisation with MeOH providing 3,7-(prop-2-yn-yloxy) flavone (**2**, 2.46 g, 7.44 mmol, 95% yield) as a white solid. Melting point: 130–132 °C; HPLC (reverse-phase, 7:3 acetonitrile—water, *R_t_* = 5.65 min.); IR (KBr) *υ* max (cm^−1^): 3274 (≡C-H), 3173 (=C-H), 2946 (-C-H), 2107 (C≡C), 1618 (C=O), 1559 (C=C); ^1^H NMR (400.14 MHz, DMSO-d6) *δ*: 8.09 (2H, m, H-2′, H-6′), 8.02 (1H, d, *J* = 8.9 Hz, H-5), 7.58 (3H, m, H-3′, H-4′, H-5′), 7.36 (1H, d, *J* = 2.3 Hz, H-8), 7.14 (1H, dd, *J* = 8.9, 2.4 Hz, H-6), 5.00 (2H, d, *J* = 2.4 Hz, H-1‴), 4.97 (2H, d, *J* = 2.5 Hz, H-1″), 3.68 (1H, t, *J* = 2.4 Hz, H-3‴), 3.47 (1H, t, *J* = 2.4 Hz, H-3″); ^13^C NMR (100.63 MHz, DMSO-d6) *δ*: 173.2 (C-4), 161.7 (C-7), 156.3 (C-8a), 155.5 (C-2), 137.8 (C-3), 130.8 (C-4′), 130.4 (C-1′), 128.5 (C-2′, C-3′, C-5′, C-6′), 126.4 (C-5), 117.5 (C-4a), 115.2 (C-6), 101.9 (C-8), 79.3 (C-3‴), 79.1 (C-3″), 78.7 (C-2″), 78.3 (C-2‴), 58.7 (C-1″), 56.3 (C-1‴). HRMS (ESI^+^) *m*/*z* calculated for C_21_H_14_O_4_ [M + H^+^] 331.096, found 331.096.

#### 3.2.2. Synthesis of 2,3,4,6 tetra-O-Acetyl-β-D-glucopyranosyl Azide (**4**)

Acetobromo-*α*-D-glucose (**3**, 500 mg, 1.22 mmol) was dissolved in acetone (5 mL). Sodium azide (98.81 mg, 1.52 mmol, 1.25 equiv) was dissolved in water (3 mL), added to the reaction mixture, and left stirring at room temperature. After 4 h, the starting material was completely consumed, and only one spot, with the same Rf as the previously synthesised 2,3,4,6 tetra-O-Acetyl-*β*-D-glucopyranosyl azide, was observed (normal-phase analytical TLC, 6:4 n-Hexane—AcOEt, *R_f_* = 0.45). The mixture was concentrated until the acetone completely evaporated and a white solid precipitated from the water. The solid was filtered and washed with water to provide 2,3,4,6 tetra-O-Acetyl-*β*-D-glucopyranosyl azide (**4**, 322.6 mg, 864.14 µmol, 71% yield). Melting point: 120–124 °C; IR (KBr) *υ* max (cm^−1^): 2970 and 2910 (-C-H aliphatic), 2119 (-N_3_), 1755 (C=O), 1241, and 1213 (C-O); ^1^H NMR (300.13 MHz, CDCl_3_) *δ*: 5.22 (1H, t, *J* = 9.5 Hz, H-3), 5.12 (1H, t, *J* = 9.8 Hz, H-4), 4.95 (1H, t, *J* = 9.1 Hz, H-2), 4.64 (1H, d, *J* = 8.9 Hz, H-1), 4.27 (1H, dd, *J* = 12.5, 4.7 Hz, H-6a), 4.16 (1H, dd, *J* = 12.5, 2.3 Hz, H-6b), 3.79 (1H, ddd, *J* = 9.9, 4.7, 2.3 Hz, H-5), 2.10 (3H, s, COC*H*_3_), 2.07 (3H, s, COC*H*_3_), 2.03 (3H, s, COC*H*_3_), 2.01 (3H, s, COC*H*_3_); ^13^C NMR (75.48 MHz, CDCl_3_) *δ*: 170.6 (*C*OCH3), 170.1 (*C*OCH3), 169.3 (*C*OCH3), 169.2 (*C*OCH3), 87.9 (C-1), 74.0 (C-5), 72.6 (C-3), 70.6 (C-2), 67.8 (C-4), 61.6 (C-6), 20.6 (4 CO*C*H3). HRMS (ESI+) *m/z* calculated for C_14_H_19_N_3_O_9_ [M + Na^+^] 396.102, found 396.094.

#### 3.2.3. Synthesis of 3,7-((2,3,4,6-tetra-O-Acetyl-β-D-glucopyranosyl-1H-1,2,3-triazole-4-yl) methoxy) flavone (**5a**)

3,7-(prop-2-yn-yloxy) flavone (**2**, 400 mg, 1.21 mmol) and 2,3,4,6-tetra-O-Acetyl-b-D-glucopyranosyl azide (**4**, 949.29 mg, 2.54 mmol; 2 equiv/alkyne) were dissolved in THF (16 mL). After solubilisation, an aqueous mixture (4 mL) of sodium ascorbate (1.92 g, 9.69 mmol, 4 equiv/alkyne) and CuSO_4_ (773.04 mg, 4.84 mmol, 2 equiv/alkyne) was added to the solution. The mixture was protected from light with aluminium foil and left stirring at room temperature. After 24 h, the starting material **2** was completely consumed (normal-phase analytical TLC, 5:5 CHCl_3_–Acetone). The mixture was filtered and washed with THF. The filtrated solution was concentrated until the THF completely evaporated and a solid precipitated from water. The solid was filtered and washed with water. The solid obtained was further purified through preparative TLC (normal-phase, 5:5 CHCl_3_–Acetone) to provide 3,7-((2,3,4,6-tetra-O-Acetyl-*β*-D-glucopyranosyl-1*H*-1,2,3-triazole-4-yl)methoxy) flavone (*R_f_* = 0.85) as a yellow solid (**5a**, 458 mg, 425.27 µmol, 35% yield). Melting point: 185–187 °C; IR (KBr) *υ* max (cm^−1^): 3468 and 3135 (=C-H triazole and Ar.), 2946 (-C-H), 1751 (C=O acetyl), 1623 (C=O flavone), 1497, and 1449 (C-C Ar.), 1227 (C-O); ^1^H NMR (400.14 MHz, DMSO-d6) *δ*: 8.62 (1H, s, -C=C*H*-triazole), 8.41 (1H, s, -C=C*H*-triazole), 8.04 (1H, d, *J* = 8.9 Hz, H-5), 8.02–7.93 (2H, m, H-2′, H-6′), 7.56–7.47 (3H, m, H-3′, H-4′, H-5′), 7.43 (1H, d, *J* = 2.4 Hz, H-8), 7.15 (1H, dd, *J* = 8.9 and 2.3 Hz, H-6), 6.39 (1H, d, *J* = 9.2 Hz, H-1″), 6.31 (1H, d, *J* = 9.2 Hz, H-1″), 5.72–5.59 (2H, dt, *J* = 18.4 and 9.3 Hz, 2 H-2″), 5.59–5.50 (2H, dd, *J* = 9.6 and 9.5 Hz, 2 H-3″), 5.36 (2H, t, *J* = 13.4 Hz, -O-C*H*_2_-triazole), 5.27 (2H, td, *J* = 12.3 and 5.9 Hz, -O-C*H*_2_-triazole), 5.22–5.11 (2H, td, *J* = 9.8 and 5.3 Hz, 2 H-4″), 4.42–4.30 (2H, dddd, *J* = 11.9, 10.1, 5.3 and 2.6 Hz, 2 H-5‴), 4.13 (2H, dd, *J* = 12.6 and 5.4 Hz, 2 H-6″a), 4.08 (2H, dd, *J* = 13.0 and 2.4 Hz, 2 H-6″b), 2.03 (6H, s, 2 COC*H*_3_), 2.00 (6H, d, *J* = 6.7 Hz, 2 COC*H*_3_), 1.96 (6H, s, 2 COC*H*_3_), 1.77–1.68 (6H, m, 2 COC*H*_3_); ^13^C NMR (100.63 MHz, DMSO-d6) *δ*: 173.3 (C-4), 169.9 (2 *C*OCH_3_), 169.5 (2 *C*OCH_3_), 169.3 (2 *C*OCH_3_), 168.3 (2 *C*OCH_3_), 162.3 (C-7), 156.4 (C-8a), 154.9 (C-2), 143.1 (-*C*=CH-triazole), 142.6 (-*C*=CH-triazole), 138.5 (C-3), 130.6 (C-4′), 130.3 (C-1′), 128.3 (C-2′, C-3′, C-5′, C-6′), 126.4 (C-5), 123.8 (2 -C=*C*H-triazole), 117.5 (C-4a), 115.1 (C-6), 101.6 (C-8), 83.8 (2 C-1″), 73.2 (2 C-5″), 72.1 (2 C-3″), 70.0 (2 C-2″), 67.5 (2 C-4″), 63.9 (-O-*C*H_2_-triazole), 61.7 (-O-*C*H_2_-triazole, 2 C-6″), 20.3 (6 CO*C*H_3_), 19.7 (2 CO*C*H_3_); HRMS (ESI^+^) *m*/*z* calculated for C_49_H_52_N_6_O_22_ [M + Na^+^] 1099.303, found 1099.288.

#### 3.2.4. Synthesis of 3,7-((2-Acetamido-3,4,6-tetra-O-Acetyl-2-deoxy-β-D-glucopyranosyl-1H-1,2,3-triazole-4-yl)methoxy) flavone (**5b**)

3,7-(prop-2-yn-yloxy) flavone (**2**, 250 mg, 716.80 µmol) was dissolved in THF (12 mL) with 2-acetamido-3,4,6-tetra-O-Acetyl-2-deoxy-*β*-D-glucopyranosyl azide (**AG931**, 619.92 mg, 1.66 mmol, 1.10 equiv/alkyne). After solubilisation, an aqueous mixture (3 mL) of CuSO_4_ (438.15 mg, 3.03 mmol, 2 equiv/alkyne) and sodium ascorbate (1.20 g, 6.05 mmol, 4 equiv/alkyne) was added to the solution. The mixture was protected from light with aluminium foil and left stirring at room temperature. After 2 h, the starting material **2** was completely consumed (normal-phase analytical TLC, 5:5 CHCl_3_—Acetone). The mixture was filtered and washed with THF. The filtrated solution was concentrated until the THF completely evaporated and a solid precipitated from water. The solid was filtered and washed with water. The solid obtained was further purified through preparative TLC (normal-phase, 5:5 CHCl_3_—Acetone) to provide 3,7-((2-acetamido-3,4,6-tetra-*O*-Acetyl-2-deoxy-*β*-D-glucopyranosyl-1H-1,2,3-triazole-4-yl)methoxy) flavone (*R_f_* = 0.28) as a white solid (**5b**, 232 mg, 215.81 µmol, 29% yield). Melting point: 267–270 °C; IR (KBr) *υ* max (cm^−1^): 3347 and 3077 (=C-H triazole and Ar.), 2958 (-C-H), 1747 (C=O acetyl), 1670 (C=O flavone), 1621 (C=O amide), 1540 (C=C triazole and Ar.), 1449 (C-C Ar.), 1232 (C-O); ^1^H NMR (400.14 MHz, DMSO-d6) *δ*: 8.49 (1H, s, -C=C*H*-triazole), 8.28 (1H, s, -C=C*H*-triazole), 8.08 (1H, d, *J* = 9.2 Hz, H-5,), 8.04 (2H, d, *J* = 9.0 Hz, 2 -N*H*-COCH_3_), 8.00–7.96 (2H, m, H-2′, H-6′), 7.57–7.49 (3H, m, H-3′, H-4′, H-5′), 7.43 (1H, d, *J* = 2.4 Hz, H-8), 7.15 (1H, dd, *J* = 8.9 and 2.4 Hz, H-6), 6.14 (1H, d, *J* = 10.0 Hz, H-1″), 6.09 (1H, d, *J* = 10.0 Hz, H-1″), 5.39–5.30 (4H, m, 2 H-3″), 5.25 (2H, d, *J* = 1.9 Hz, 2 -O-C*H*_2_-triazole), 5.10 (2H, dt, *J* = 9.7 and 2.9 Hz, 2 H-4″), 4.60 (2H, dq, *J* = 19.5 and 9.9 Hz, 2 H-2″), 4.27–4.19 (2H, dddd, *J* = 10.7, 10.7, 5.0 and 2.3, 2 H-5″), 4.19–4.02 (4H, m, 2 H-6″, H-6‴), 2.02–2.00 (6H, m, 2 COC*H*_3_), 1.99 (6H, s, 2 COC*H*_3_), 1.94 (6H, s, 2 COC*H*_3_), 1.54 (6H, d, *J* = 6.5 Hz, 2 -NH-COC*H*_3_); ^13^C NMR (100.63 MHz, DMSO-d6) *δ*: 173.3 (C-4), 169.9 (2 *C*OCH_3_), 169.5 (2 *C*OCH_3_), 169.3 (2 *C*OCH_3_, 2 -NH-*C*OCH_3_), 162.4 (C-7), 156.5 (C-8a), 155.1 (C-2), 142.7 (-*C*=CH-triazole), 142.1 (-*C*=CH-triazole), 138.6 (C-3), 130.6 (C-4′), 130.3 (C-1′), 128.3 (C-2′, C-3′, C-5′, C-6′), 126.4 (C-5), 123.7 (2 -C=*C*H-triazole), 117.6 (C-4a), 115.1 (C-6), 101.6 (C-8), 84.7 (2 C-1″), 73.3 (2 C-5″), 72.3 (2 C-3″), 67.9 (2 C-4″), 64.1 (2 -O-*C*H_2_-triazole), 61.7 (2 C-6″), 51.9 (2 C-2″), 22.2 (2 -NH-CO*C*H_3_), 20.4 (6 CO*C*H_3_); HRMS (ESI^+^) *m*/*z* calculated for C_49_H_54_N_8_O_20_ [M + Na^+^] 1097.335, found 1097.319.

#### 3.2.5. Synthesis of 3,7-((2-Azidoethyl 2,3,4,6-tri-O-acetyl-2-deoxy-β-D-glucopyranosyl-1H-1,2,3-triazole-4-yl)methoxy) flavone (**5c**)

3,7-(prop-2-yn-yloxy) flavone (**2**, 200 mg, 605.44 µmol) was dissolved in THF (8 mL) with 2-azidoethyl 2,3,4,6-tri-*O*-acetyl-*β*-D-glucopyranosyl azide (**AG904**, 555.92 mg, 1.33 mmol, 1.10 equiv/alkyne). After solubilisation, an aqueous mixture (2 mL) of CuSO_4_ (386.52 mg, 2.42 mmol, 2 equiv/alkyne) and sodium ascorbate (959.38 mg, 4.84 mmol, 4 equiv/alkyne) was added to the solution. The mixture was protected from light with aluminium foil and left stirring at room temperature. After 2 h, the starting material **2** was completely consumed (normal-phase analytical TLC, 5:5 CHCl_3_–Acetone). The mixture was filtered and washed with THF. The filtrated solution was concentrated until the THF completely evaporated. The water solution was extracted with AcOEt. The organic layer was dried with Na_2_SO_2_, followed by crystallisation from MeOH/CH_2_Cl_2_. The crystallisation provided 3,7-((2-azidoethyl 2,3,4,6-tri-*O*-acetyl-2-deoxy-*β*-D-glucopyranosyl-1H-1,2,3-triazole-4-yl)methoxy) flavone (*R_f_* = 0.65) as a yellow solid (**5c,** 170 mg, 145.91 µmol, 24% yield). Melting point: 190–193 °C; IR (KBr) *υ* max (cm^−1^): 3470 and 3134 (=C-H triazole and Ar.), 2961 (-C-H), 1750 (C=O acetyl), 1637 (C=O flavone), 1621 (C=C triazole and Ar.), 1501 and 1448 (C-C Ar.), 1227 (C-O); ^1^H NMR (400.14 MHz, DMSO-d6) *δ*: 8.16 (1H, s, -C=C*H*-triazole), 8.04 (1H, d, *J* = 8.9 Hz, H-5), 7.97 (2H, m, H-2′, H-6′), 7.88 (1H, s, -C=C*H*-triazole), 7.59–7.48 (3H, m, H-3’, H-4′, H-5′), 7.46 (1H, d, *J* = 2.4 Hz, H-8), 7.13 (1H, dd, *J* = 8.9 and 2.3 Hz, H-6), 5.31 (2H, s, -O-C*H*_2_-triazole), 5.22 (2H, t, *J* = 9.6 Hz, 2 H-2″), 5.21 (2 H, s, -O-C*H*_2_-triazole), 4.89 (2H, td, *J* = 9.7 and 2.9 Hz, 2 H-3″), 4.81 (2H, dd, *J* = 14.7 and 8.0 Hz, 2 H-1″), 4.72 (2H, td, *J* = 9.5 and 8.0 Hz, 2 H-4″), 4.58 (2H, m, -O-CH_2_-C*H*_2_-triazole), 4.46 (2H, m, -O-CH_2_-C*H*_2_-triazole), 4.22–3.78 (10H, m, 2 H-5″, 2 H-6″, 2 -O-C*H*_2_-CH_2_-triazole), 2.01 (6H, d, *J* = 4.1 Hz, 2 COC*H*_3_), 1.97 (6H, s, 2 COC*H*_3_), 1.91 (6H, s, 2 COC*H*_3_), 1.86 (6H, d, *J* = 12.8 Hz, 2 COC*H*_3_); ^13^C NMR (100.63 MHz, DMSO-d6) *δ*: 173.3 (C-4), 170.0 (2 *C*OCH_3_), 169.5 (2 *C*OCH_3_), 169.2 (2 *C*OCH_3_), 168.9 (2 *C*OCH_3_), 162.5 (C-7), 156.5 (C-8a), 155.3 (C-2), 142.2 (-*C*=CH-triazole), 141.6 (-*C*=CH-triazole), 138.5 (C-3), 130.6 (C-4′), 130.3 (C-1′), 128.3 (C-2′, C-3′, C-5′, C-6′), 126.4 (C-5), 125.2 (-C=*C*H-triazole), 124.9 (-C=*C*H-triazole), 117.5 (C-**4a**), 115.1 (C-6), 101.4 (C-8), 99.1 (2 C-1″), 71.8 (2 C-2″), 70.5 (2 C-4″), 68.0 (2 C-3″), 67.4 (2 -O-*C*H_2_-CH_2_-triazole, 2 C-5″), 64.2 (-O-*C*H_2_-triazole), 61.8 (-O-*C*H_2_-triazole), 61.6 (2 C-6″), 49.3 (-O-CH_2_-*C*H_2_-triazole), 49.1 (-O-CH_2_-*C*H_2_-triazole), 20.1 (8 CO*C*H_3_); HRMS (ESI^+^) *m*/*z* calculated for C_53_H_60_N_6_O_24_ [M + Na^+^] 1187.356, found 1187.338.

### 3.3. Nanotechnology

#### 3.3.1. Preparation of Empty and Loaded PMs

Self-assembled structures were prepared by a solvent displacement method with PEG_45_PLA_106_ diblock copolymer (**PEGPLA**), and the targeted PMs were prepared by the co-assembly of **PEGPLA** diblock copolymer and T7-functionalized PEG_20_PLA_106_ (**T7-PEGPLA**) in 0.1 mol% of **T7-PEGPLA** to 99.9 mol% **PEGPLA** ratio. Briefly, 20 mg of copolymers was solubilized in DMF (and 2 mg of each compound for loaded PMs). A total of 600 μL of the solution was then injected into 1.4 mL of MiliQ water (eq. 30% *v*/*v*) at a flow rate of 100 μL/min under magnetic stirring at 500 rpm using a syringe pump (New Era Pump Systems, Inc., Farmingdale, NY, USA). The solution containing the self-assembled nanostructures was then dialyzed using a 3.5 kDa dialysis membrane (Cellu.Sept^®^, membrane filtration products Inc., Seguin, TX, USA) over-night in 1000 mL of purified water.

#### 3.3.2. Particle Size, Zeta Potential, and Polydispersion Index

The samples were prepared by adding 250 μL of PM sample in 1000 μL of purified water and analysed using Dynamic Light Scattering (DLS) with a ZETASIZER Pro apparatus (Malvern Panalytical, Worcestershire, UK). The collected data, mean diameter, zeta potential, and polydispersion index (PDI) were obtained by the ZS XPLORER 3.3.1.5 software (Malvern Panalytical, Worcestershire, UK) and were expressed as mean ± standard deviation throughout the work.

#### 3.3.3. Negative-Staining Transmission Electronic Microscopy (TEM)

For negative staining, 5 µL of samples were mounted on Formvar/carbon film-coated 300 mesh nickel grids (Electron Microscopy Sciences, Hatfield, PA, USA) and left standing for 2 min. The liquid in excess was removed with filter paper, and 5 µL of 2% uranyl acetate was added onto the grids and left standing for 30 s, after which the liquid in excess was removed with filter paper. The visualisation was performed on a JEOL JEM 1400 TEM at 120 kV (Tokyo, Japan), and the images were digitally recorded using a PHURONA digital camera (Munich, Germany). The TEM was performed at the HEMS core facility at i3S, University of Porto, Portugal, with the assistance of Sofia Pacheco and Rui Fernandes.

#### 3.3.4. Stability Study

The stability investigation was conducted on the PMs. The samples were intermittently assessed for the mean diameter and PDI at determined time points post-manufacturing, while the formulations were preserved at a temperature of 4 °C.

#### 3.3.5. Entrapment Efficiency

The obtained formulations were centrifuged (24,000× *g* for 25 min) (Model 5804, Eppendorf, Hauppauge, NY, USA), and the supernatant was discarded. The pellet was resuspended in 1 mL of acetonitrile, and it was filtered (0.45 μm PTFE filter, OlimPeak^®^, Teknokroma, Barcelona, Spain) to obtain the compounds entrapped amount sample. The obtained samples were then evaluated by HPLC (Dionex UltiMateTM 3000 from Thermo Fisher Scientific, Bremen, Germany) equipped with a quaternary pump and a quaternary Variable Wavelength Detector. Chromeleon 7.2 software was used for data acquisition.

The chromatographic conditions included a commercially available AcclaimTM 120 C18 (100 × 4.6 mm) column with a particle size of 5 μm from Thermo Fisher Scientific (Bremen, Germany). The optimised mobile phase for **5a**–**5c** consisted of 50:50 H_2_O—Acetonitrile, following an isocratic flow of 1.0 mL/min for 15 min. The injection volume was 10 μL, and the detection was performed at 259 nm. An individual calibration curve for all compounds was prepared at concentrations of 100.0, 50.0, 25.0, 12.5, 6.13 and 3.1 µM in triplicate. The drug concentration in the pellet was obtained by interpolation from the calibration curve. The theoretical concentration of the compound was calculated by considering the amount of compound placed during the production of the PMs and the dilutions performed throughout the procedure, and the direct method of the encapsulation efficiency (*EE*%) can be calculated using Equation (1):

(1) EE (%)=Entrapped Amount/Total Amount,

where the total amount is the initial concentration of the compound in the formulation (μM) and the entrapped amount is the final concentration of the compound in the pellet (μM).

#### 3.3.6. MTT Assays

The human tumour cell lines U-251 (glioblastoma astrocytoma) and L929 (fibroblasts) were grown in DMEM, both supplemented with 10% FBS and 1% Pen-Strep. All cell lines were maintained at 37 °C in a 5% CO_2_ humidified atmosphere (Hera Cell, Heraeus). The L929 and U-251 cell lines were cultivated as reported by Mendes et al. [13,14]. The passages used for the L929 cell line were P18–P21 and P23–P27, and the U-251 cell line was P + 5–P + 8. 

For the 3-(4,5-dimethylthiazol-2-yl)-2,5-diphenyltetrazolium bromide (MTT) assay, the cells were seeded in 96-well plates (5.00 × 10^4^ cells/well for L929 and 1.00 × 10^4^ cells/well for U-251) and exposed to either different concentrations of compounds dissolved in medium with 5% DMSO or different concentrations of the PMs formulations for 24 h. The quantification of viable cells was conducted by introducing the MTT reagent and allowing for an incubation period of 2 h at a temperature of 37 °C.

To dissolve the crystals, DMSO was employed. The following controls were used: DMEM, DMEM with 5% DMSO, and DMEM with 1% Triton. The absorbance was read at 570 nm and 630 nm for background subtraction, and the results of cell viability of all wells were expressed as CV%.

### 3.4. Statistical Analysis

The results are shown as the mean ± standard deviation of three or five batches of the same formulation. The results of mean diameter, EE, and cell viability were statistically analysed using ANOVA (two-way). The differences between the groups for ANOVA were compared with a post hoc test (Dunnett’s test). The significance was set at *p* < 0.05.

In the figures, different asterisks (*) in the same sample represent significant differences: *p*-value > 0.05; * (One asterisk): *p*-value < 0.05; ** (Two asterisks): *p*-value < 0.01; *** (Three asterisks): *p*-value < 0.001; **** (Four asterisks): *p*-value < 0.0001.

All the statistical analyses were performed using GraphPad Prism 10 for macOS (Version 10.1.1, 21 November 2023).

## 4. Conclusions

In this study, three acetoglycosyl triazole-linked flavonoids were successfully synthesised using click chemistry. These flavonoids were encapsulated within functionalized T7 peptide PMs, utilising the same click chemistry strategy, for local delivery and to ensure the solubility of the synthesised flavones. The results demonstrated significant reductions in the viability of human glioblastoma U-251 cells at given concentrations, namely the PM system containing compound **5c**, reducing cell viability by more than 50% across nearly all tested concentrations. All PMs showed a mean size between 120 and 180 nm, which constitutes a favourable size range for nanoparticle extravasation in vascular tissues. Also, the entrapment efficacy of this **5c**-PM (39%) underlines the potential of this particles in local high dosage therapies.

Therefore, the developed “click-on-click” DDS shows promising potential for targeting not only the world’s most aggressive and deadly brain cancers but also in modern therapy. The rapid and precise synthesis enabled by click chemistry not only paves the way for the development of highly targeted therapies but also opens up opportunities to revolutionise cancer treatment. This DDS represents an exciting step toward providing more efficient and localised therapeutic options for GBM patients, a cancer notoriously resistant to conventional treatments.

We can further refine and expand the applicability of this system, offering the potential for a more effective, targeted approach to GBM therapy. By addressing these goals, this study can provide an innovative and targeted approach to GBM treatment, moving towards the possibility of transforming glioblastoma from a terminal to a more manageable health condition.

## Figures and Tables

**Table 1:** The mean diameter, PDI, and zeta potential (ζ) of loaded and functionalised PMs prepared by the solvent displacement method (mean ± SD, *n* = 3).

PMs Formulation	Mean Diameter (nm)	PDI	ζ (mV)
0 mol% **T7-PEGPLA** (control)	119.4 ± 3.352	0.0882	−28.53
0.1 mol% **T7-PEGPLA** (control)	122.7 ± 2.701	0.0489	−27.70
0.1 mol% **T7-PEGPLA**-**5a**	184.6 ± 27.89	0.3877	−24.66
0.1 mol% **T7-PEGPLA**-**5b**	161.6 ± 30.64	0.2687	−20.71
0.1 mol% **T7-PEGPLA**-**5c**	153.2 ± 1.900	0.0688	−28.88

## Data Availability

Data sharing is not applicable.

## Supplementary Materials

The following supporting information can be downloaded at https://www.mdpi.com/article/10.3390/pharmaceutics17060771/s1. Figure S1: ^1^H NMR (**A**) and ^13^C NMR (**B**) spectra of **5a**; Figure S2: HSQC (**A**) and HMBC (**B**) spectra of **5a**; Figure S3: HMRS (**A**) and IR (**B**) spectra of **5a**; Figure S4: ^1^H NMR (**A**) and ^13^C NMR (**B**) spectra of **5b**; Figure S5: HSQC (**A**) and HMBC (**B**) spectra of **5b**; Figure S6: HMRS (**A**) and IR (**B**) spectra of **5b**; Figure S7: ^1^H NMR (**A**) and ^13^C NMR (**B**) spectra of **5c**; Figure S8: HSQC (**A**) and HMBC (**B**) spectra of **5c**; Figure S9: HMRS (**A**) and IR (**B**) spectra of **5c**.
