# Supplementary material for: Click on Click: Click-Flavone Glycosides Encapsulated in Click-Functionalised Polymersomes for Glioblastoma Therapy
Source: Pharmaceutics. 2025 Jun 12;17(6):771. doi: 10.3390/pharmaceutics17060771 (PMC12196103; doi:10.3390/pharmaceutics17060771)
Supplement: Supplementary file 1 [file pharmaceutics-17-00771-s001.zip › pharmaceutics-3648307-supplementary.pdf]

Supplementary Material

Click on Click: Click-Flavone Glycosides Encapsulated in Click-Functionalised Polymersomes for Glioblastoma Therapy

Nuno M. Saraiva [1,2], Ana Alves [3,4*], Ana Isabel Barbosa [5], Andreia Marinho [5], Salette Reis [5], Marta Correia-da-Silva [1,2 *], Paulo C. Costa [3,4]

1 LQOF—Laboratory of Organic and Pharmaceutical Chemistry, Department of Chemical Sciences, Faculty of Pharmacy, University of Porto, Rua Jorge de Viterbo Ferreira 228, 4050-313 Porto, Portugal
2 CIIMAR—Interdisciplinary Center of Marine and Environmental Research, University of Porto, Terminal dos Cruzeiros do Porto de Leixões, Avenida General Norton de Matos, S/N, 4450-208 Matosinhos, Portugal
3 UCIBIO—Applied Molecular Biosciences Unit, MedTech-Laboratory of Pharmaceutical Technology, Faculty of Pharmacy, University of Porto, Rua Jorge de Viterbo Ferreira 228, 4050-313 Porto, Portugal
4 Associate Laboratory i4HB, Institute for Health and Bioeconomy, Faculty of Pharmacy, University of Porto, Rua Jorge de Viterbo Ferreira 228, 4050-313 Porto, Portugal
5 LAQV, REQUIMTE-Associated Laboratory for Green Chemistry, Department of Chemical Sciences, Faculty of Pharmacy, University of Porto, Rua Viterbo Ferreira 228, 4050-313 Porto, Portugal.
* Correspondence: m_correiadasilva@ff.up.pt, anadaniela92@hotmail.com

Figure S1. **1D** NMR Spectra of Compound **5a**

(A)

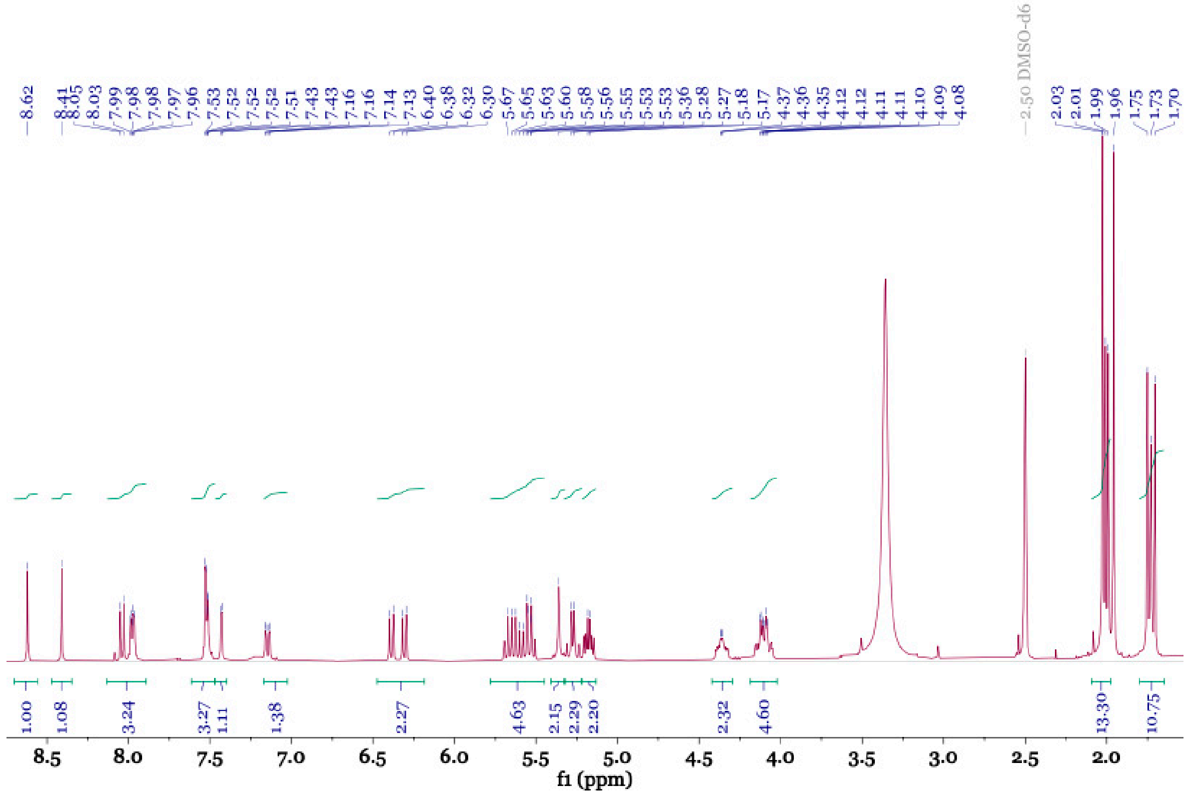

(B)

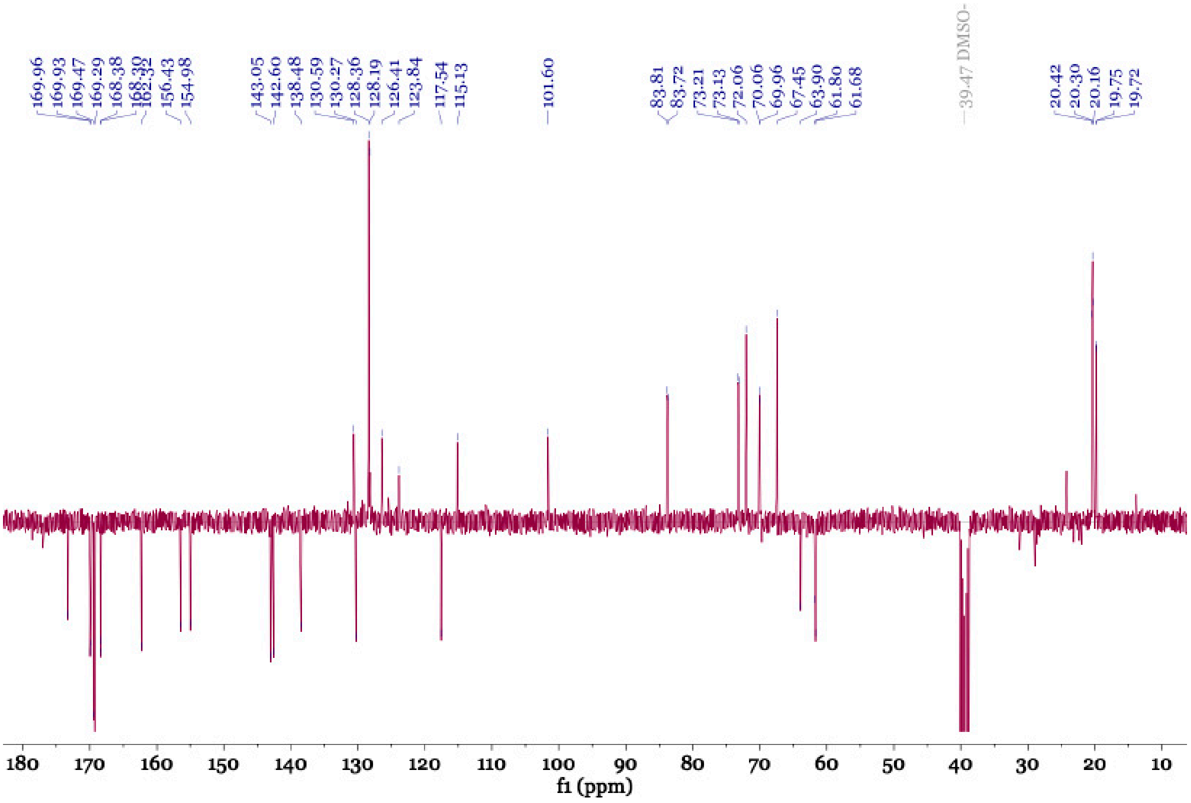

Figure S1 – ^{1}H NMR **(A)** and ^{13}C NMR **(B)** spectra of **5a.**

Figure S2. **2D** NMR Spectra of Compound **5a**

(A)

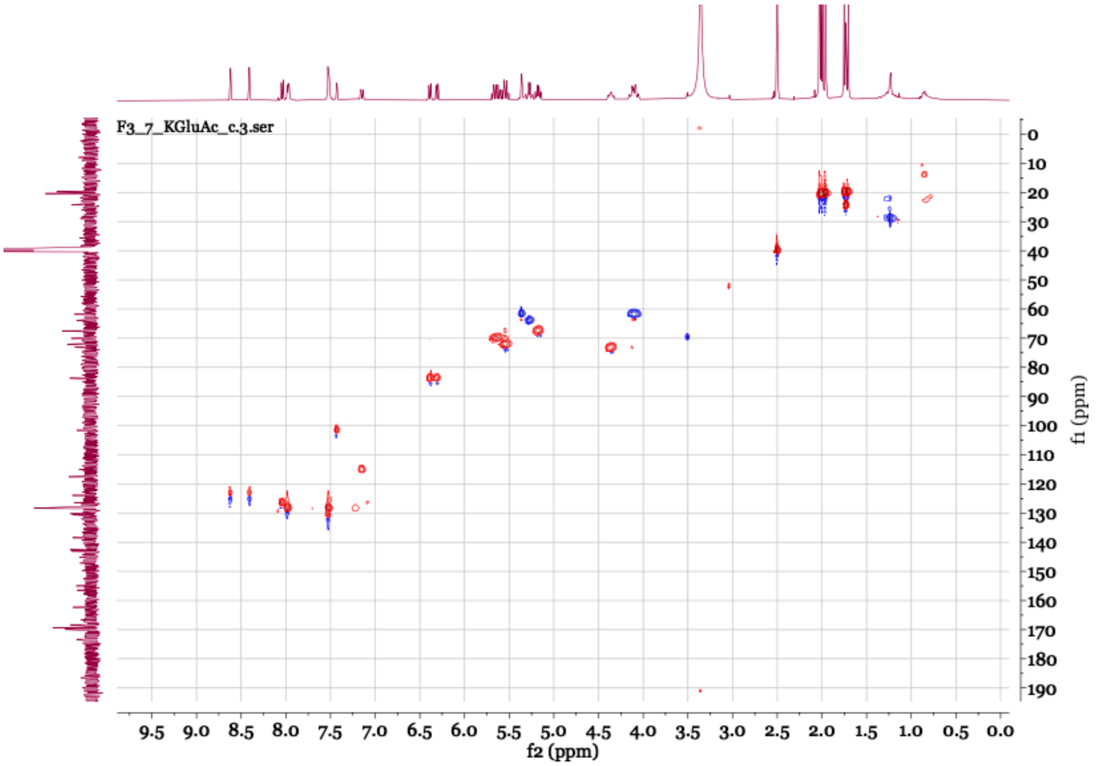

(B)

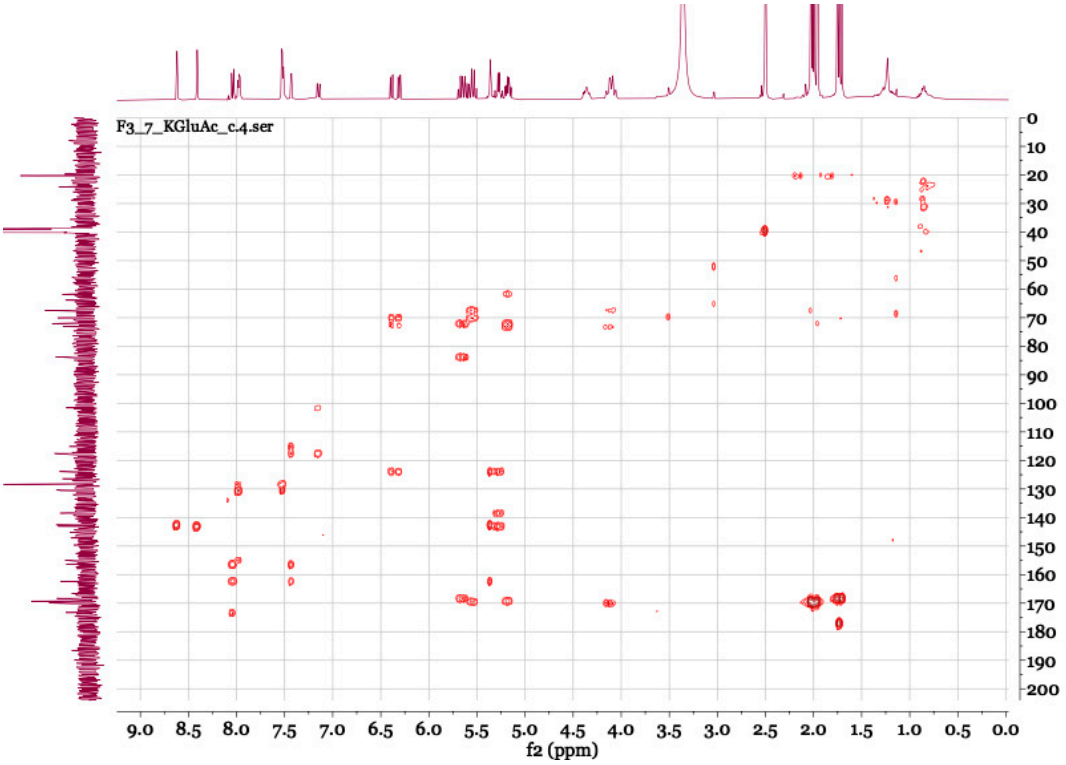

Figure S2 – HSQC **(A)** and HMBC **(B)** spectra of **5a.**

Figure S3. HMRS and IR Spectra of Compound **5a**

(A)

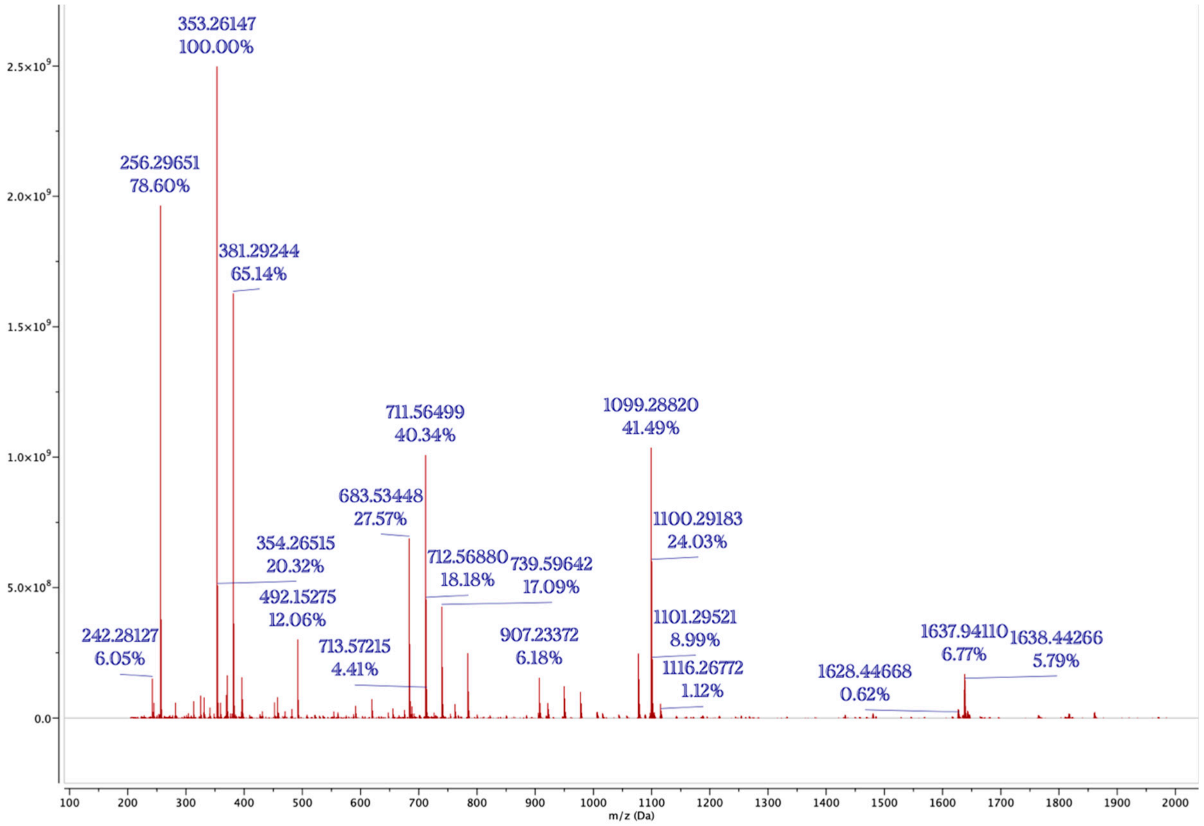

(B)

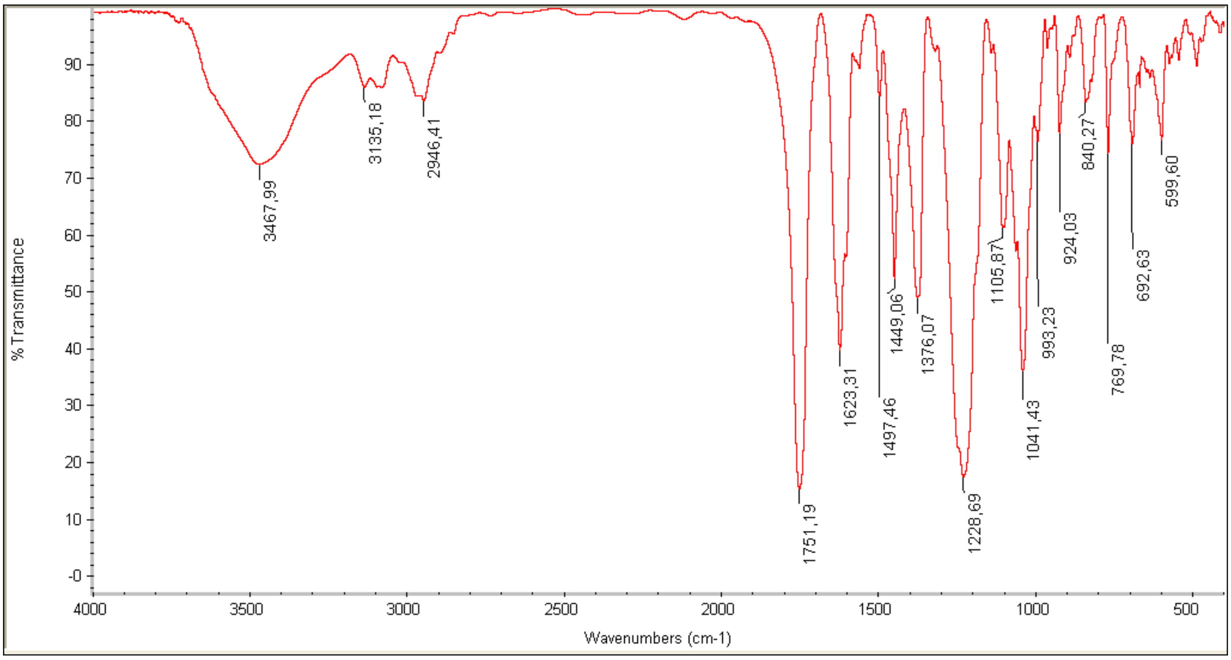

Figure S3 – HMRS **(A)** and IR **(B)** spectra of **5a.**

Figure S4. **1D** NMR Spectra of Compound **5b**

(A)

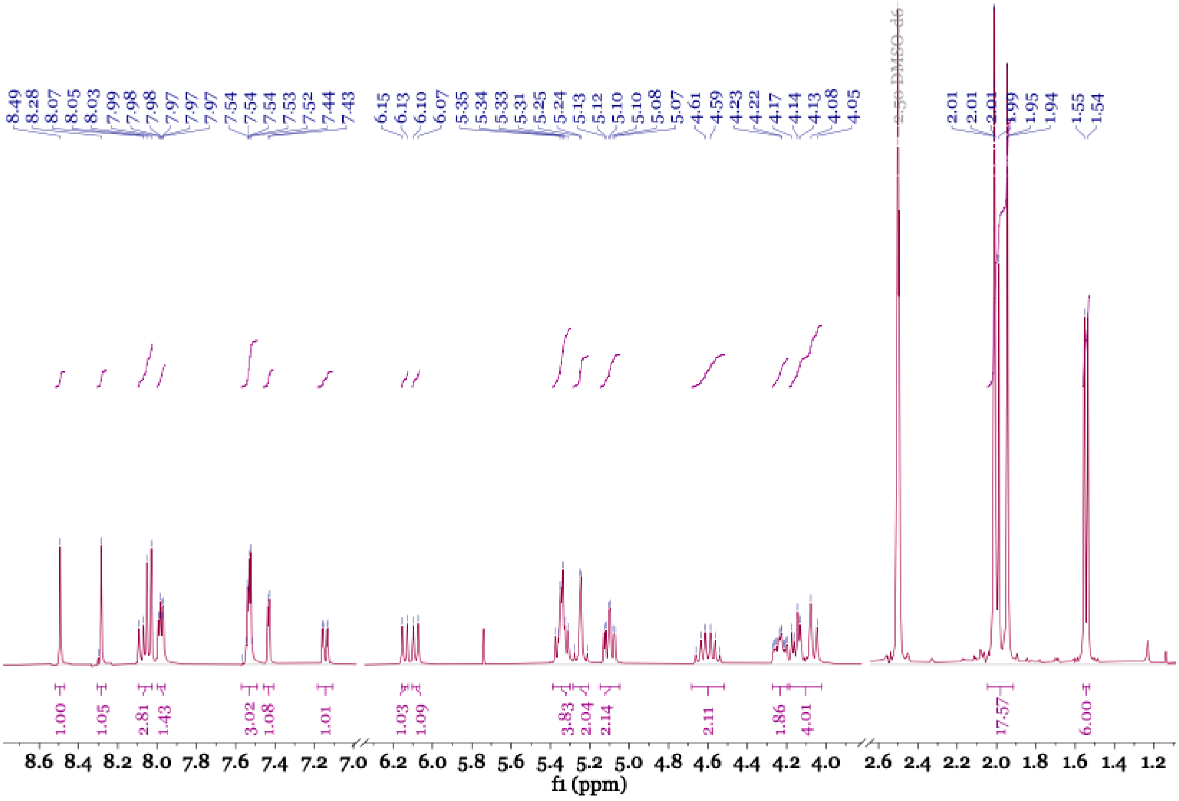

(B)

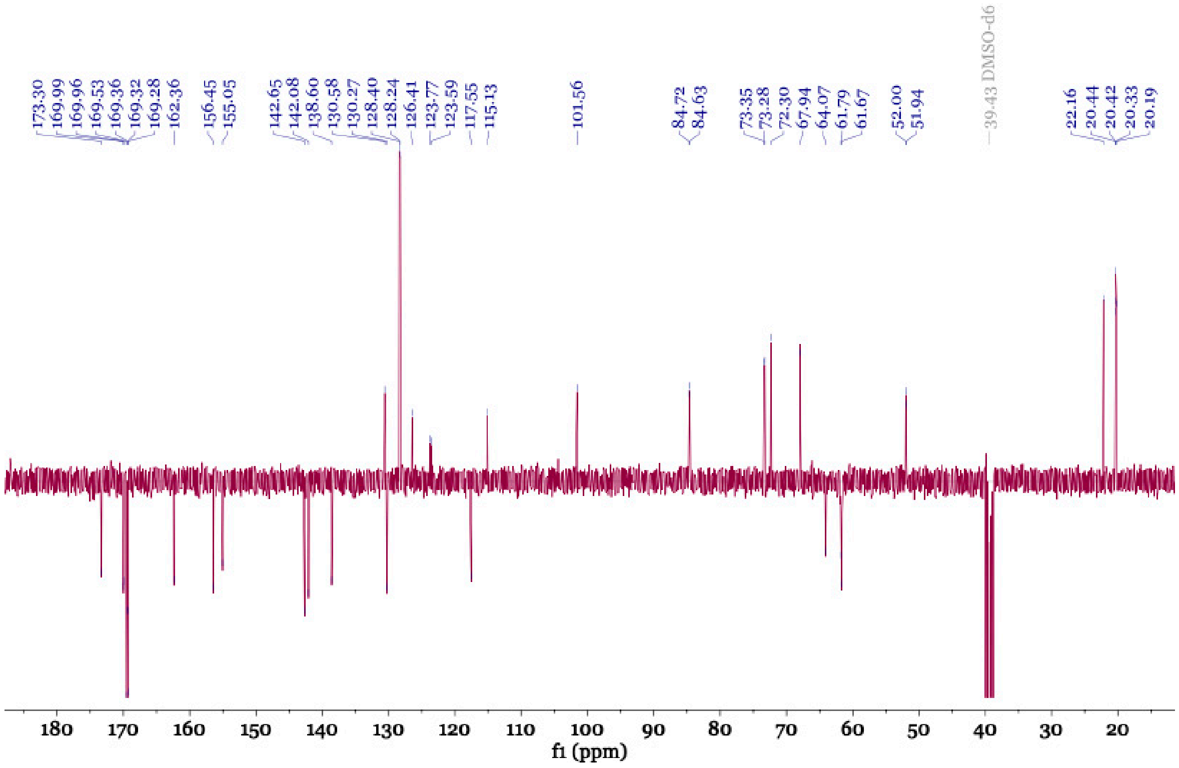

Figure S4 – ^{1}H NMR **(A)** and ^{13}C NMR **(B)** spectra of **5b.**

Figure S5. 2D NMR Spectra of Compound **5b**

(A)

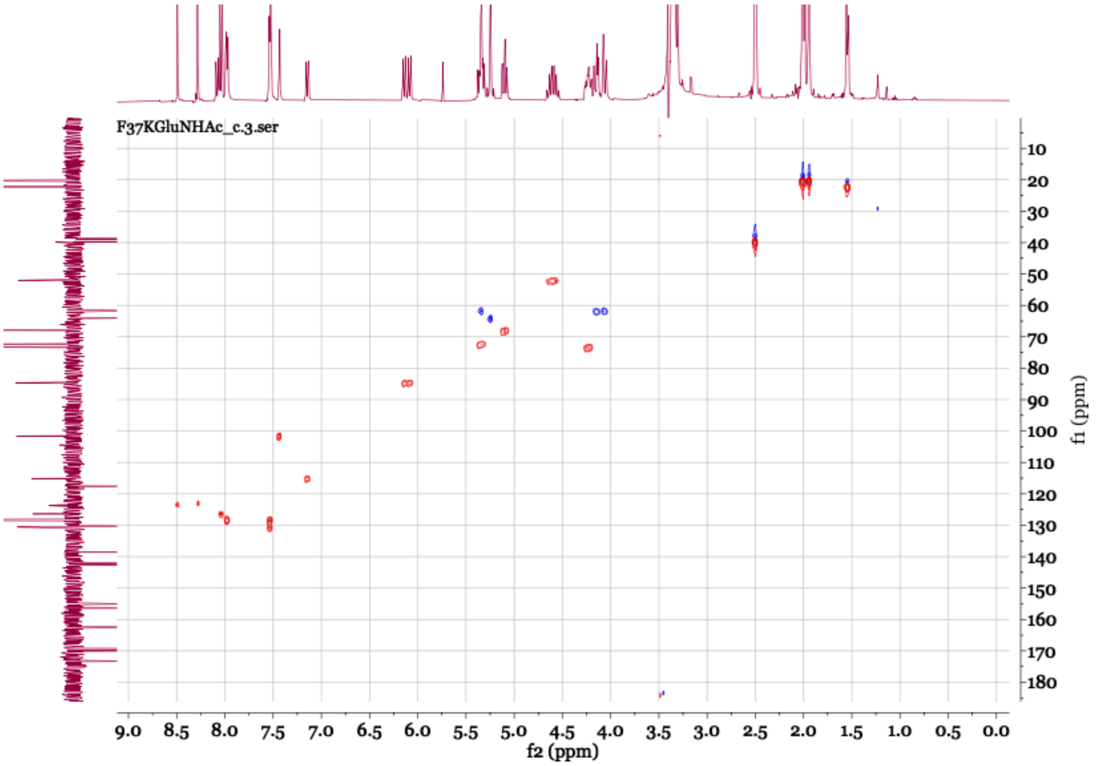

(B)

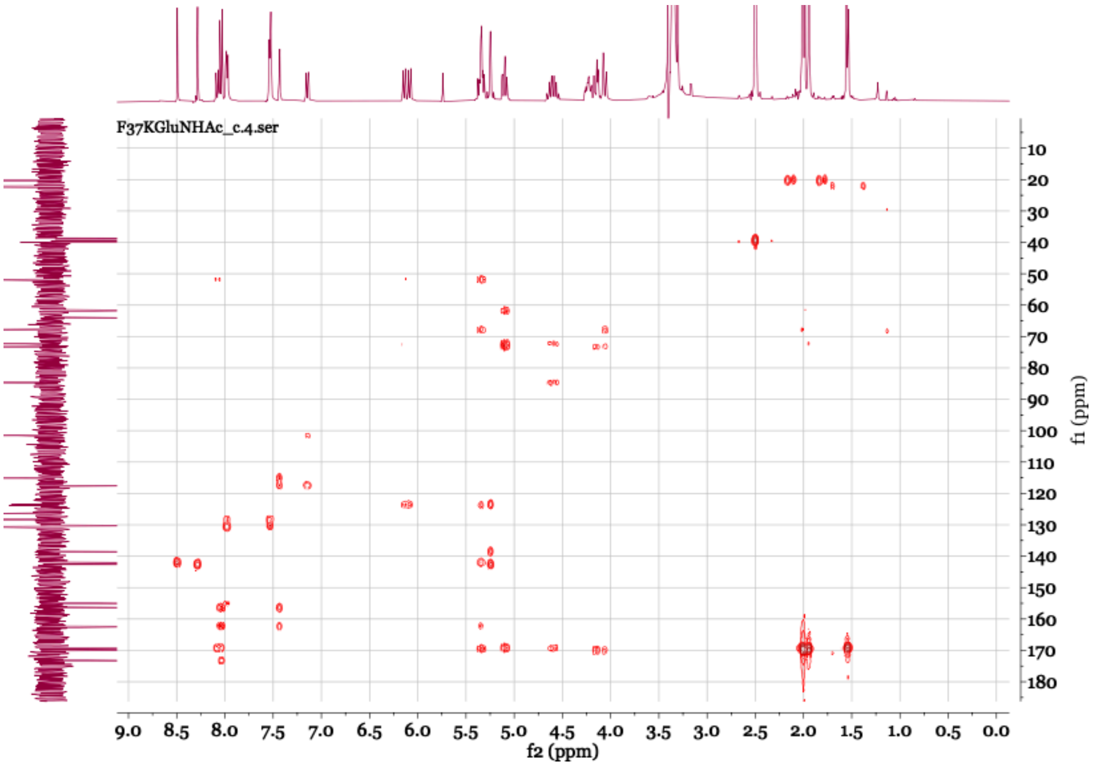

Figure S5 – HSQC **(A)** and HMBC **(B)** spectra of **5b.**

Figure S6. HMRS and IR Spectra of Compound **5b**

(A)

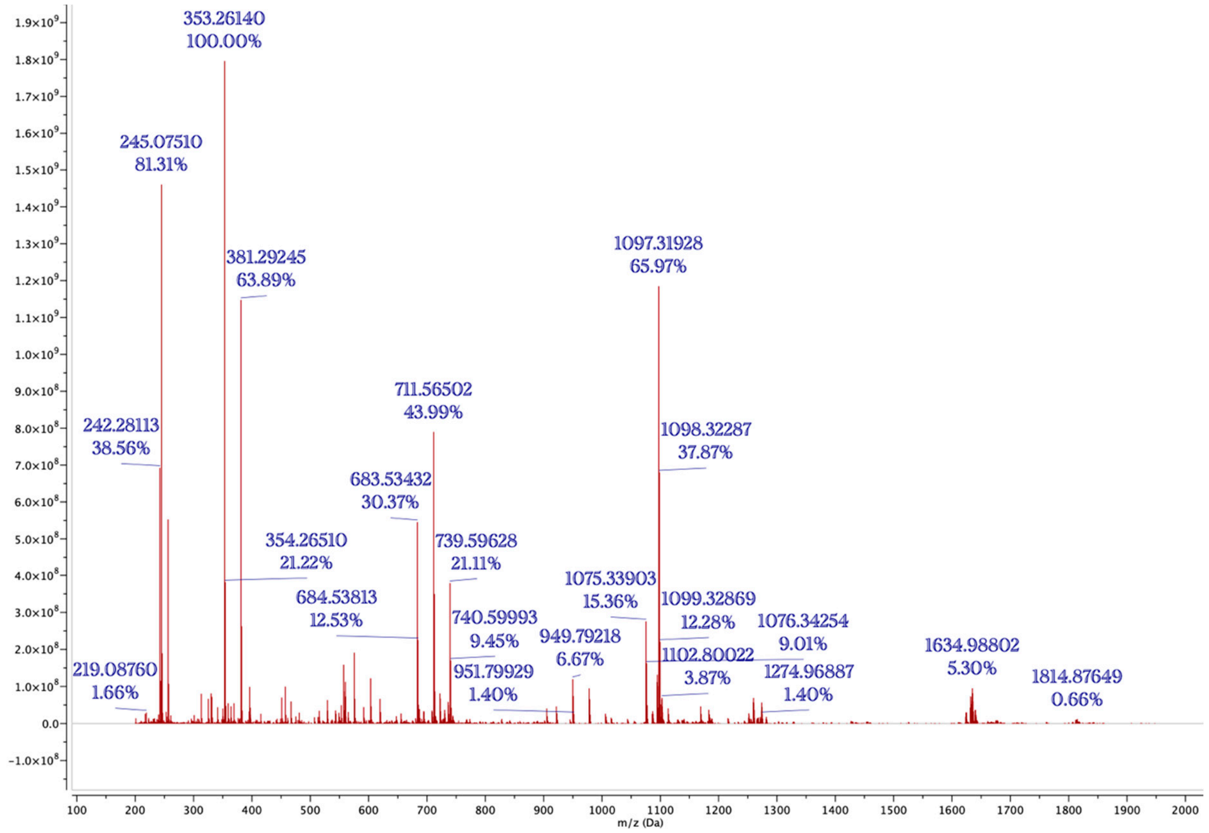

(B)

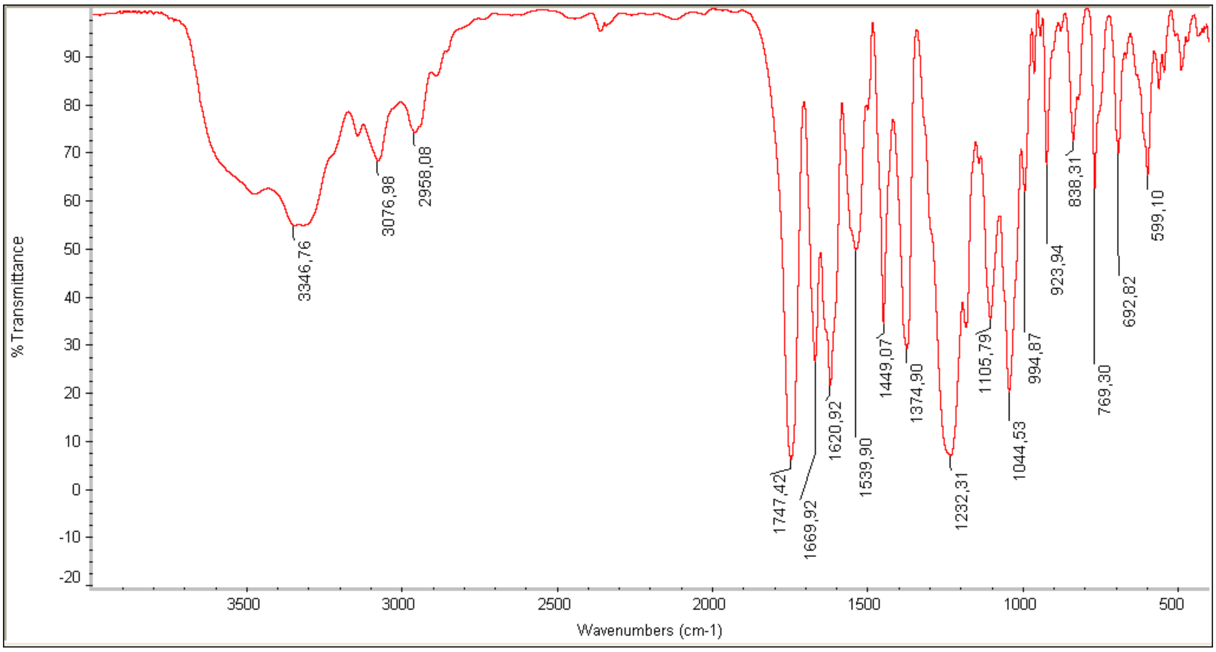

Figure S6 – HMRS **(A)** and IR **(B)** spectra of **5b.**

Figure S7. **1D** NMR Spectra of Compound **5c**

(A)

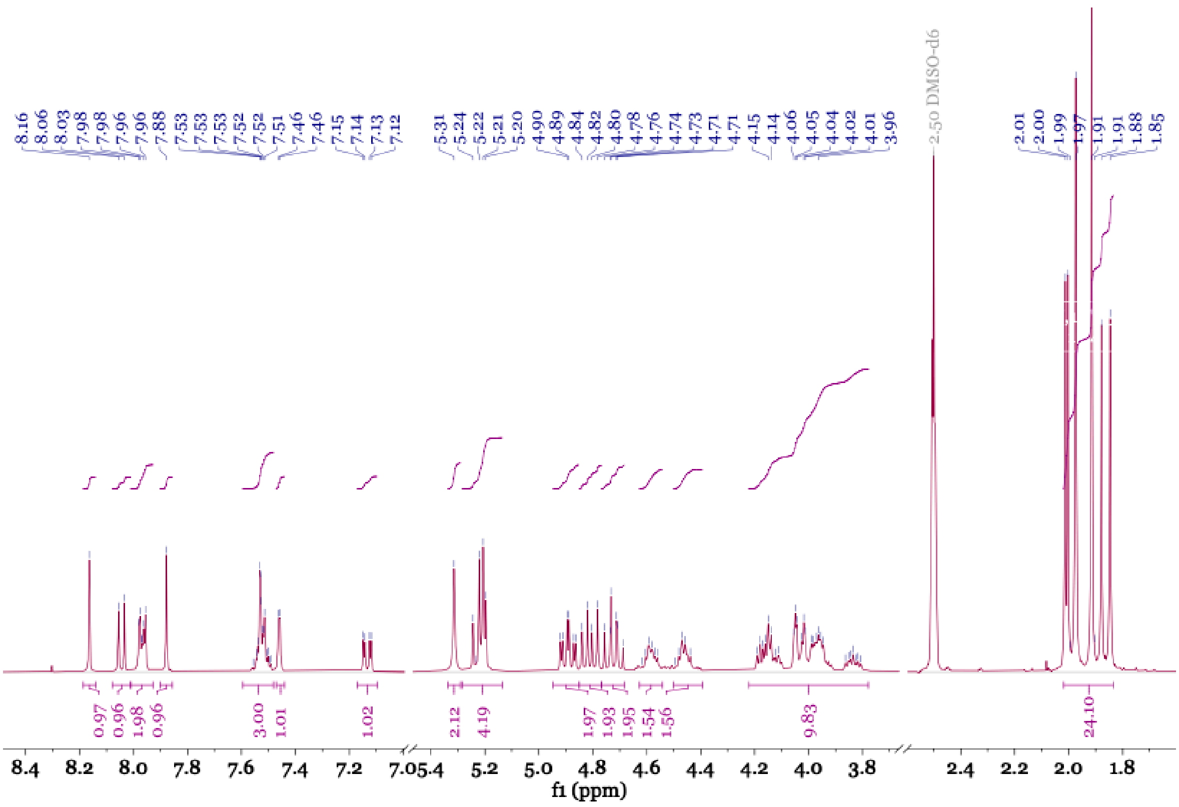

(B)

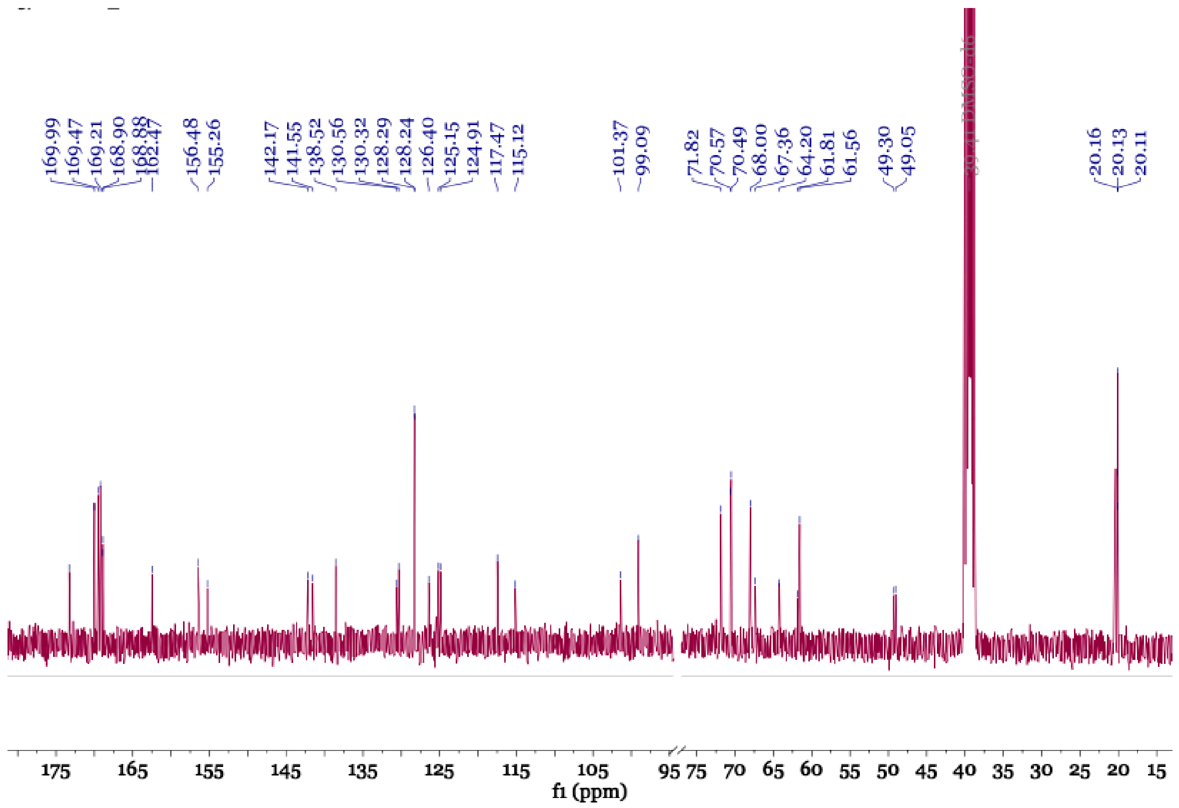

Figure S7 – ^{1}H NMR **(A)** and ^{13}C NMR **(B)** spectra of **5c.**

Figure S8. **2D** NMR Spectra of Compound **5c**

(A)

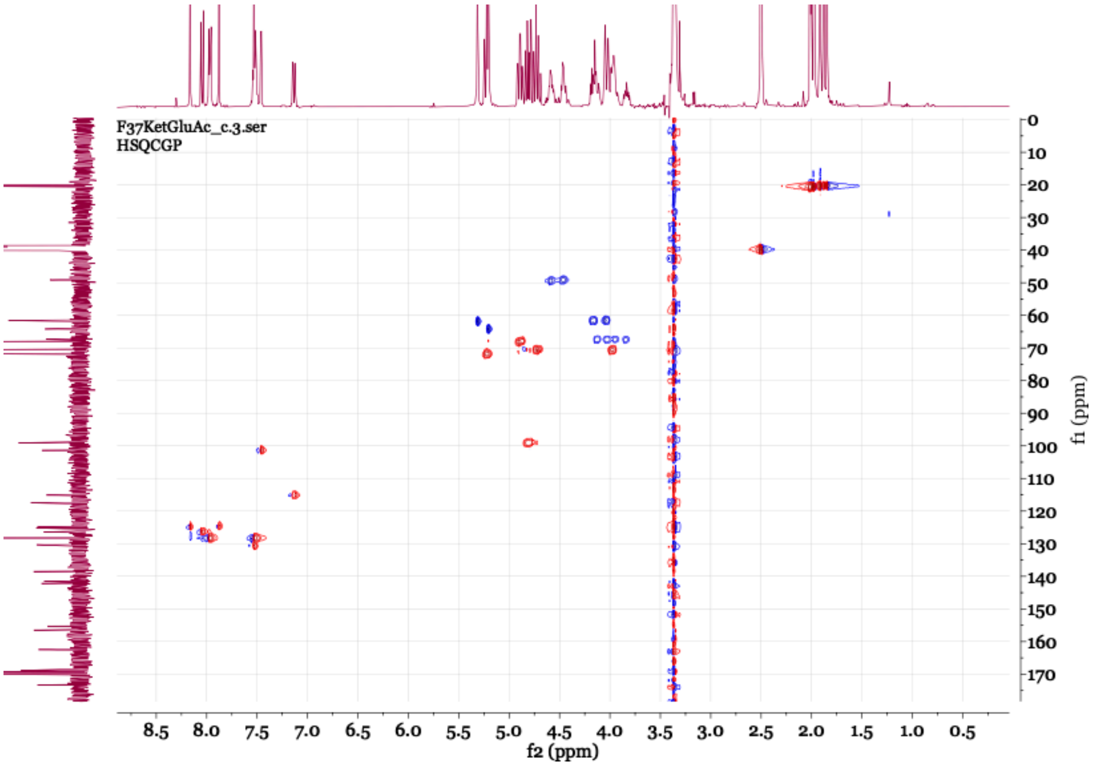

(B)

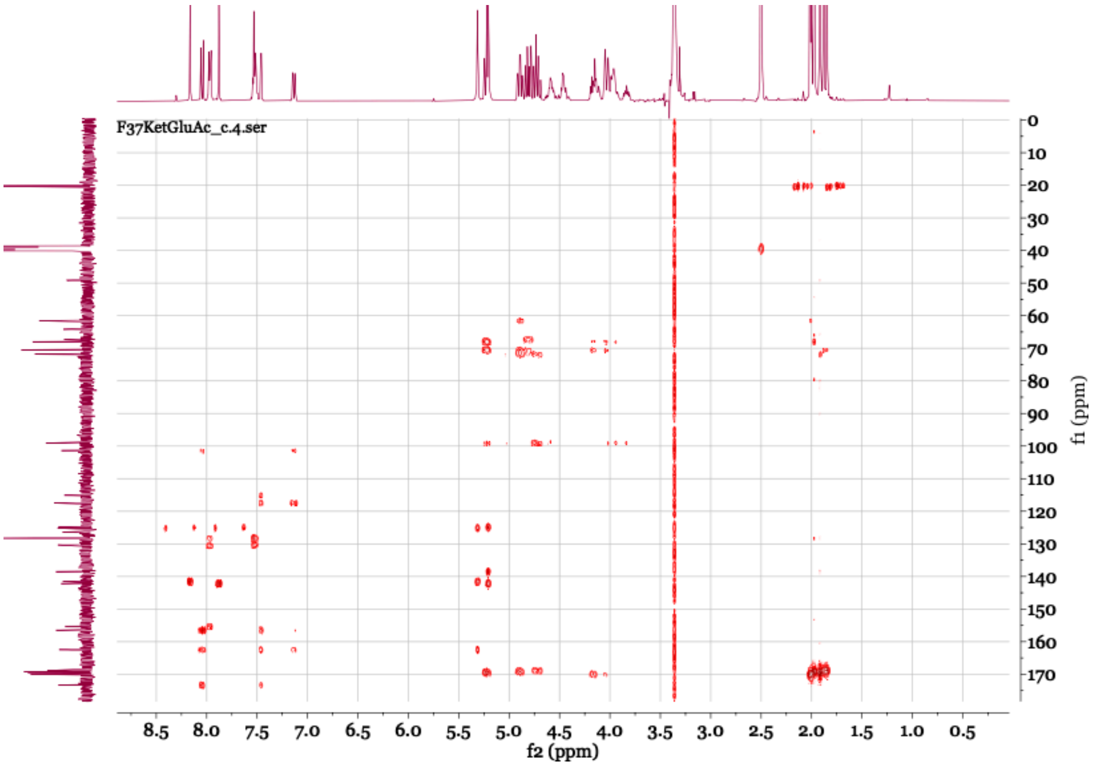

Figure S8 – HSQC **(A)** and HMBC **(B)** spectra of **5c.**

Figure S9. HMRS and IR Spectra of Compound **5c**

(A)

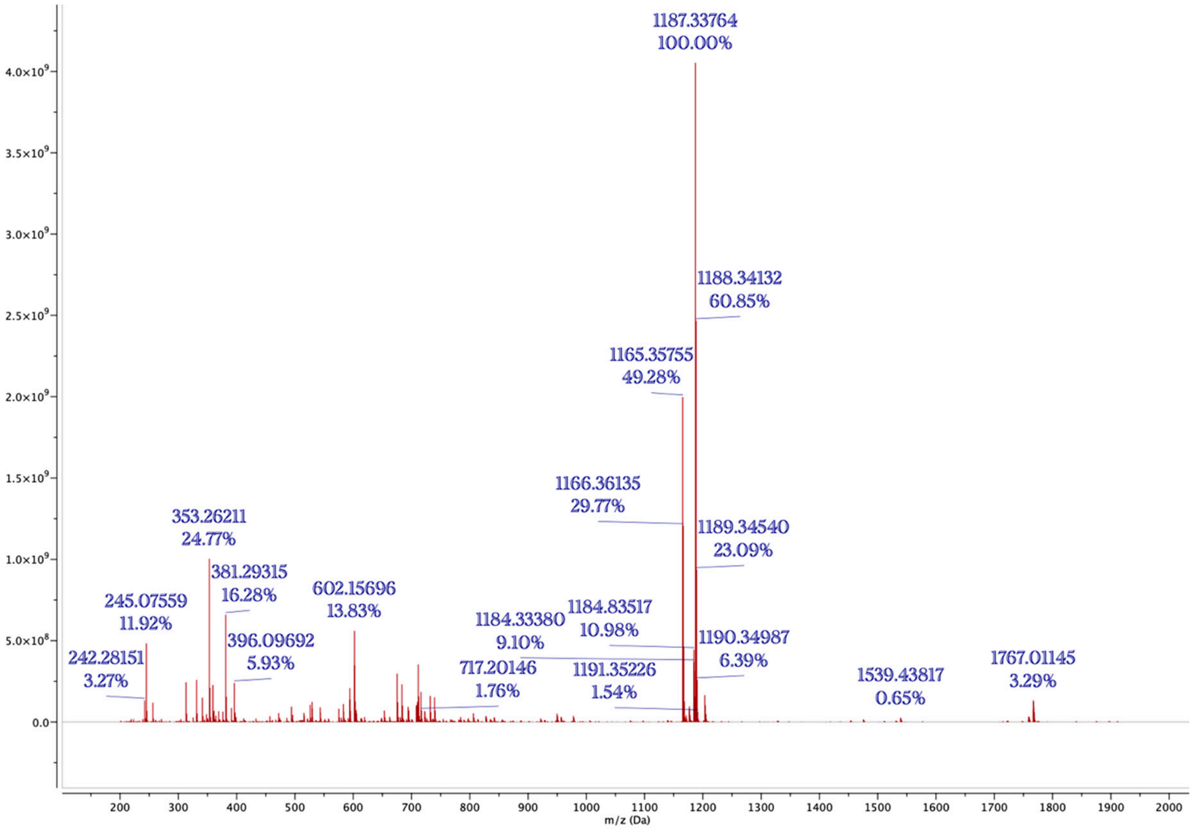

(B)

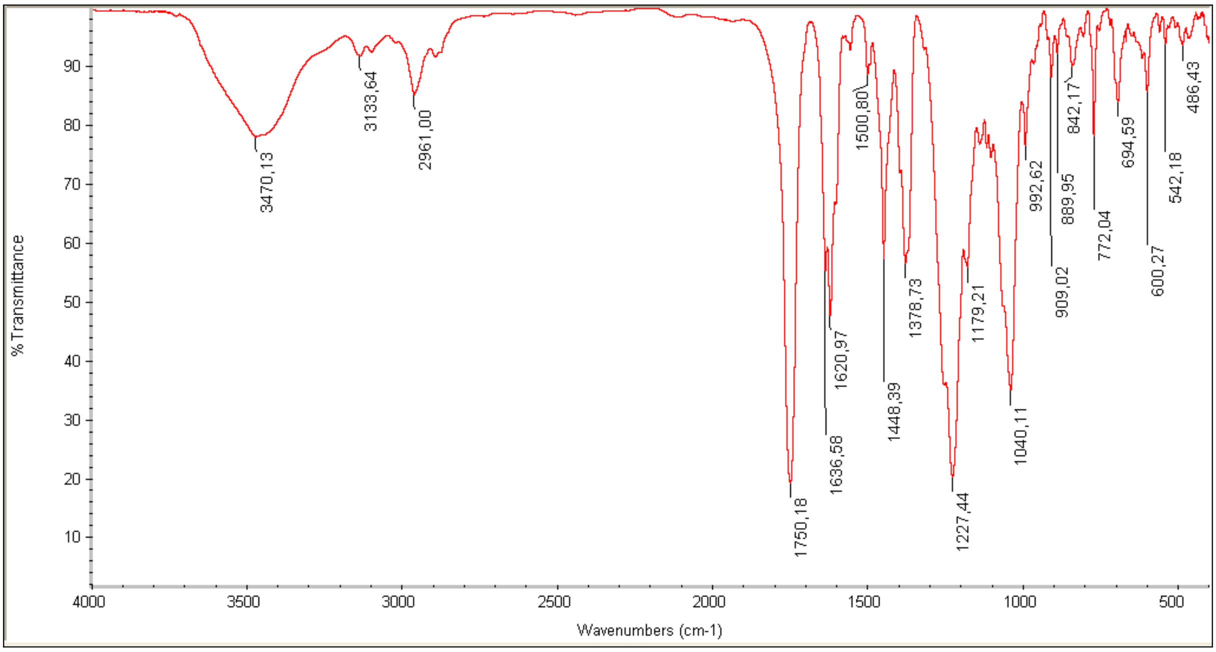

Figure S9 – HMRS **(A)** and IR **(B)** spectra of **5c.**
